# Targeting ferroptosis and cuproptosis in gastrointestinal cancers: molecular mechanisms, metabolic vulnerabilities, and therapeutic interventions

**DOI:** 10.1186/s43556-025-00347-7

**Published:** 2025-11-07

**Authors:** Yang Zhang, Yixiang Gu, Ming Zhan, Linhua Yang, Hui Wang

**Affiliations:** 1 Department of Biliary-Pancreatic Surgery, Renji Hospital, Shanghai Jiao Tong University School of Medicine, Shanghai, 200011 China; 2 Shanghai Key Laboratory of Biliary Tract Disease, Renji Hospital, Shanghai Jiao Tong University School of Medicine, Shanghai, 200011 China; 3 Shanghai Key Laboratory of Cancer System Regulation and Clinical Translation, Shanghai, 201800 China; 4 The Core Laboratory in Medical Center of Clinical Research, Shanghai Ninth People’s Hospital, State Key Laboratory of Medical Genomics, Shanghai Jiao Tong University School of Medicine, Shanghai, 200125 China; 5 Department of Systems Biology, Beckman Research Institute, City of Hope, Monrovia, CA 91016 USA

**Keywords:** Gastrointestinal cancer, Ferroptosis, Cuproptosis, Metabolic vulnerability, Therapeutic intervention, Crosstalk

## Abstract

Gastrointestinal (GI) malignancies represent a significant global health burden, characterized by high mortality rates and profound resistance to conventional therapies. This necessitates the exploration of novel therapeutic vulnerabilities, and two recently discovered forms of regulated cell death, ferroptosis and cuproptosis, offer promising metabolism-centered strategies. Ferroptosis is a non-apoptotic pathway driven by iron-dependent lipid peroxidation, canonically suppressed by the glutathione peroxidase 4 (GPX4) axis. In contrast, cuproptosis is a distinct process wherein excess copper induces lethal proteotoxic stress through direct binding to lipoylated components of the tricarboxylic acid (TCA) cycle. Critically, these pathways are not mutually exclusive; instead, they are intricately connected through shared molecular nodes and metabolic dependencies, including redox homeostasis, key signaling proteins, and mitochondrial integrity. This review systematically examines the molecular crosstalk between ferroptosis and cuproptosis, highlighting the synergistic potential of their co-activation as a powerful anti-cancer strategy in GI tumors. We systematically evaluate both preclinical evidence and clinical studies for therapeutic interventions, ranging from small-molecule inducers to advanced nanoplatforms and immunotherapy combinations. Furthermore, we discuss the pressing challenges of identifying predictive biomarkers for patient stratification and overcoming adaptive resistance. Ultimately, deciphering the ferroptosis-cuproptosis nexus holds immense potential to unlock a new paradigm of synergistic therapies, paving the way for more effective clinical management of GI malignancies.

## Introduction

Gastrointestinal (GI) malignancies, which encompass hepatocellular carcinoma (HCC), biliary tract cancer (BTC), gastric cancer (GC), colorectal cancer (CRC), pancreatic ductal adenocarcinoma (PDAC), and esophageal cancer (ESCC), constitute one of the leading causes of cancer-related mortality worldwide [1–4]. According to the latest global cancer statistics, there were over 4.8 million new cases and 3.4 million deaths from GI tumors in 2022, collectively imposing a severe global health burden [5, 6]. Among them, CRC is the third most common cancer and the second leading cause of cancer death globally. In contrast, liver and stomach cancers are the third and fifth leading causes of cancer-related mortality, respectively [7, 8]. Epidemiological data indicate that while the incidence of some GI tumors, such as GC, has declined globally, the rates for other types, including CRC and pancreatic cancer, are projected to rise significantly in the coming decades [4, 9–11]. Despite advances in diagnosis and treatment, the overall survival rates for patients with GI tumors remain unsatisfactory due to tumor heterogeneity, frequent diagnosis at advanced stages, and the prevalence of primary or acquired resistance to conventional chemoradiotherapy, targeted therapy, and even immunotherapy [12–14].

Consequently, targeting non-apoptotic regulated cell death (RCD) pathways ​represents a promising strategy to circumvent​ therapy resistance in GI cancers. This is particularly true for non-apoptotic forms of cell death that can circumvent the resistance mechanisms associated with traditional apoptosis pathways. [15, 16]. Recently, two metal-dependent forms of RCD, driven by metal ion dyshomeostasis, have emerged as critical mediators of cell death: ferroptosis and cuproptosis. These forms reveal specific metabolic dependencies that can be exploited in oncology [17]. Ferroptosis is an iron-dependent form of cell death driven by lipid peroxidation [18]. Its core execution process involves extensive lipid peroxidation, which contains polyunsaturated fatty acids (PUFAs) in cell membranes, and is tightly regulated by the System Xc⁻–glutathione (GSH)–glutathione peroxidase 4 (GPX4) axis, supported by parallel antioxidant pathways, such as the FSP1-CoQ10 axis [19, 20]. In contrast, cuproptosis is a unique, mitochondria-dependent form of cell death in which excess copper ions directly bind to lipoylated proteins of the tricarboxylic acid (TCA) cycle, such as dihydrolipoamide S-acetyltransferase (DLAT). This binding leads to their aggregation, the loss of iron-sulfur cluster proteins, and ultimately, lethal proteotoxic stress [21, 22].

In various GI tumors, these two cell death pathways are intricately linked to tumor-specific metabolic reprogramming, aberrant signaling pathways, and therapeutic resistance [23, 24]. Studies have shown that complex crosstalk exists between the molecular mechanisms of ferroptosis and cuproptosis, providing a rationale for developing synergistic therapeutic strategies [25, 26]. Pharmacological induction of these death pathways ​has yielded significant tumor suppression​ in preclinical models, particularly through the use of small-molecule inducers and advanced nanodelivery systems. This is especially true when these approaches are combined with immunotherapy, radiotherapy, or chemotherapy, where they can enhance efficacy through mechanisms such as inducing immunogenic cell death (ICD) [15, 27]. This review aims to systematically elucidate the core molecular mechanisms of ferroptosis and cuproptosis, explore their specific regulatory networks and resulting metabolic vulnerabilities in different GI tumors, comprehensively summarize therapeutic intervention strategies targeting these pathways, and discuss the prospects and challenges of their clinical translation, to provide new insights for overcoming refractory GI tumors. A thorough understanding of the core molecular machinery of these death modalities and their complex interaction network is a prerequisite for developing effective therapeutic strategies.

## Mechanistic core

### Ferroptosis: an iron-driven lipid peroxidation cascade

**Fig. 1:**
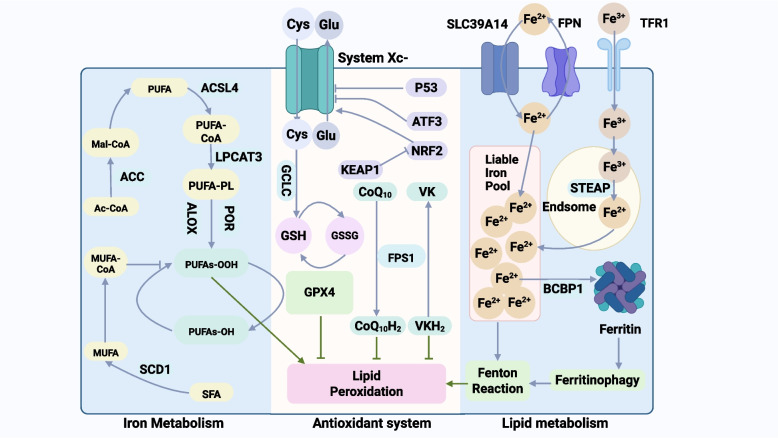
Basic mechanism of ferroptosis. Ferroptosis is an iron-dependent form of regulated cell death driven by excess lipid peroxidation. Key triggers include iron overload and GSH depletion (via cystine-import blockade by System Xc⁻ or direct GPX4 inactivation), which together lead to loss of GPX4 activity. Without GPX4, reactive oxygen species (ROS) generated by iron (Fenton chemistry) and by PUFA phospholipids in cell membranes. Important regulators of ferroptosis include the GSH/GPX4 antioxidant system; membrane lipid-metabolizing enzymes ACSL4 and LPCAT3 (which incorporate PUFAs into phospholipids, increasing their peroxidation susceptibility); and FSP1, which regenerates CoQ-10 to trap lipid radicals in parallel with GPX4. Mitochondria can amplify ferroptosis through ROS production and Fe–S cluster disruption, although ultimately ferroptosis manifests as extensive lipid oxidation and membrane damage leading to cell death. Abbreviations: ACC, acetyl-CoA carboxylase; Ac-CoA, acetyl-CoA; ACSL4, acyl-CoA synthetase long-chain family member 4; ALOX, arachidonate lipoxygenase; ATF3, activating transcription factor 3; BH4, tetrahydrobiopterin; CoQ10, coenzyme Q10 (ubiquinone); CoQ10H2, ubiquinol (reduced coenzyme Q10); Cys, cysteine; DHODH, dihydroorotate dehydrogenase; FPN, ferroportin (SLC40A1); FSP1, ferroptosis suppressor protein 1; GCH1, GTP cyclohydrolase 1; GCLC, glutamate-cysteine ligase catalytic subunit; Glu, glutamate; GPX4, glutathione peroxidase 4; GSH, glutathione; GSSG, oxidized glutathione; KEAP1, Kelch-like ECH-associated protein 1; LIP, labile iron pool; LPCAT3, lysophosphatidylcholine acyltransferase 3; LOX, lipoxygenase; Mal-CoA, malonyl-CoA; mGPX4, mitochondrial GPX4; MUFA, monounsaturated fatty acid; MUFA-CoA, monounsaturated fatty acyl-CoA; NCOA4, nuclear receptor coactivator 4; NRF2, nuclear factor erythroid 2–related factor 2; PEBP1, phosphatidylethanolamine-binding protein 1; POR, cytochrome P450 oxidoreductase; PUFA, polyunsaturated fatty acid; PUFA-CoA, polyunsaturated fatty acyl-CoA; PUFA-PL, polyunsaturated fatty acid-containing phospholipid; PUFAs-OH, hydroxylated polyunsaturated fatty acids; PUFAs-OOH, hydroperoxide forms of polyunsaturated fatty acids; ROS, reactive oxygen species; SCD1, stearoyl-CoA desaturase 1; SFA, saturated fatty acid; SLC39A14, ZIP14 (zinc transporter 14); STEAP, six-transmembrane epithelial antigen of the prostate (metalloreductase family); System Xc⁻, cystine/glutamate antiporter (SLC7A11/SLC3A2); TFR1, transferrin receptor 1; VK, vitamin K; VKH2, vitamin K hydroquinone

Ferroptosis is an iron-dependent form of regulated cell death driven by lipid peroxidation, which is morphologically, biochemically, and genetically distinct from other cell death modalities such as apoptosis, necrosis, and autophagy [18, 28]. This process is characterized by an imbalance between cellular oxidative and antioxidant systems, ultimately resulting in severe peroxidative damage to membrane phospholipids [29]. Its complex molecular regulatory network can be understood as a dynamic equilibrium between a "pro-death" substrate supply system and a "pro-survival" core detoxification system [30]. On one hand, polyunsaturated fatty acids (PUFAs) in cell membranes and the labile iron pool provide the necessary chemical foundation for ferroptosis. On the other hand, a robust intracellular antioxidant network, particularly the defense axis centered on glutathione peroxidase 4 (GPX4), constitutes the critical line of defense against ferroptosis [19]. When this defense is breached, the initiation of ferroptosis becomes inevitable. The core molecular mechanism of ferroptosis is illustrated in Fig. 1 of this review.

The initiation and execution of ferroptosis are highly dependent on two key elements of its "pro-death arm": oxidizable lipid substrates and the iron ions that catalyze the oxidative reactions [31]. First, PUFAs embedded in membrane phospholipids, especially arachidonic acid (AA) and adrenic acid (AdA), are the primary substrates for lipid peroxidation [31]. Acyl-CoA synthetase long-chain family member 4 (ACSL4) acts as the "initiator" in this process, responsible for activating these free PUFAs into their corresponding acyl-CoAs [32]. Subsequently, lysophosphatidylcholine acyltransferase 3 (LPCAT3) efficiently incorporates these activated PUFAs into membrane phospholipid molecules, a process that significantly increases the membrane's susceptibility to peroxidative damage [32]. Lipid peroxidation can be initiated through both enzymatic and non-enzymatic pathways. The enzymatic pathway is primarily mediated by iron-dependent lipoxygenases (LOXs) [30]. A pivotal study found that the scaffold protein phosphatidylethanolamine-binding protein 1 (PEBP1) can form a complex with 15-lipoxygenase (15-LO). This binding alters the substrate specificity of 15-LO, enabling it to directly catalyze the peroxidation of phosphatidylethanolamine (PE) in the membrane, thereby generating hydroperoxy-phosphatidylethanolamine (hydroperoxy-PE) and triggering the ferroptosis signal [33]. Second, central to the "iron-dependent" nature of ferroptosis, ferrous ions (Fe^2^⁺) in the labile iron pool are the key catalysts for the Fenton reaction, which generates highly reactive hydroxyl radicals. These radicals, in turn, initiate and propagate the chain reaction of lipid peroxidation [17, 18]. Cells tightly regulate the level of the labile iron pool through various mechanisms, among which the selective autophagy process known as ferritinophagy is a key upstream event promoting ferroptosis [34]. In this process, the specific cargo receptor, nuclear receptor coactivator 4 (NCOA4), recognizes and binds to the intracellular iron-storage protein complex, ferritin, thereby targeting it for degradation in the lysosome. This action releases a large amount of iron ions, greatly expanding the labile iron pool and providing an ample catalyst for the Fenton reaction [34].

In contrast, the "pro-survival arm" comprises multiple precise and hierarchical antioxidant systems designed to neutralize or repair damage caused by lipid peroxidation. The most classic and central defense axis is the System Xc⁻–glutathione (GSH)–GPX4 pathway [19, 30]. System Xc⁻, composed of the SLC7A11 and SLC3A2 subunits, is responsible for importing extracellular cystine, which is then reduced to cysteine intracellularly, providing the key raw material for GSH biosynthesis [35]. As the most abundant hydrophilic antioxidant in the cell, GSH is an essential reducing cofactor for the catalytic activity of GPX4 [19, 36]. GPX4 is a unique selenoenzyme that crucially utilizes GSH to efficiently reduce lipid hydroperoxides (PLOOH) formed on membrane phospholipids to their non-toxic corresponding lipid alcohols (PLOH), thereby directly terminating the lipid peroxidation chain reaction [37, 38]. However, the cell's defense system is not limited to this pathway. Recent studies have revealed several parallel, GSH-independent defense pathways. One important pathway is the ferroptosis suppressor protein 1 (FSP1)–coenzyme Q10 (CoQ10) axis [20, 39]. FSP1 is a membrane-localized, NAD(P)H-dependent oxidoreductase that reduces the oxidized form of CoQ10 (ubiquinone) to its reduced form, CoQH₂ (ubiquinol) [39]. As a potent lipophilic radical-trapping antioxidant, ubiquinol can directly scavenge lipid peroxy radicals within the membrane, thus inhibiting the initiation and propagation of ferroptosis independently of the GSH/GPX4 system [20]. Another parallel defense pathway is mediated by GTP cyclohydrolase 1 (GCH1) and its product, tetrahydrobiopterin (BH4) [40]. The expression of GCH1 leads to the synthesis of BH4, which not only possesses intrinsic antioxidant activity but also contributes to a robust anti-ferroptotic barrier by promoting the endogenous production of CoQ10 and regulating lipid remodeling [40].

At the mitochondrial level, this organelle plays an indispensable and complex role in the regulatory network of ferroptosis. It acts as both a signal amplifier and possesses its own independent defense systems; its dysfunction and oxidative stress are key components of the ferroptotic mechanism [41, 42]. Although the final execution site of ferroptosis is the rupture of the plasma membrane, the functional state of mitochondria profoundly influences a cell's susceptibility to ferroptosis [43, 44]. First, mitochondria are the primary sites of reactive oxygen species (ROS) in the cell [41]. During the operation of the mitochondrial electron transport chain (mtETC), superoxide anions and other ROS inevitably "leak" out, significantly exacerbating the cell's overall oxidative stress level. This synergizes with Fenton reaction-driven lipid peroxidation, potently amplifying the ferroptosis signal [29, 45]. Second, mitochondria are the crossroads of several key metabolic pathways, including the tricarboxylic acid (TCA) cycle, fatty acid β-oxidation, and the biosynthesis of iron-sulfur (Fe-S) clusters [38]. These metabolic activities are directly linked to the core elements of ferroptosis. For example, the metabolic state of the TCA cycle can impact the cellular redox balance. At the same time, dysfunction of mitochondria as the center of Fe-S cluster synthesis can directly affect iron metabolism and distribution, potentially leading to an expansion of the labile iron pool and thus promoting ferroptosis [46]. More importantly, mitochondria harbor specific ferroptosis defense mechanisms. GPX4 has its own mitochondrial isoform (mGPX4), which is specifically responsible for eliminating lipid peroxides in the inner mitochondrial membrane, thereby protecting mitochondrial structure and functional integrity [47]. Furthermore, a mitochondrial defense mechanism independent of the classic pathways revolves around dihydroorotate dehydrogenase (DHODH), which is localized to the inner mitochondrial membrane [48]. While playing its role in the de novo pyrimidine synthesis pathway, DHODH can also utilize its redox activity to reduce ubiquinone to ubiquinol on the mitochondrial membrane. This activity forms a potent local antioxidant barrier that effectively inhibits mitochondrial lipid peroxidation and protects the cell against ferroptosis [48].

### Cuproptosis: mitochondrial proteotoxicity from copper overload

**Fig. 2:**
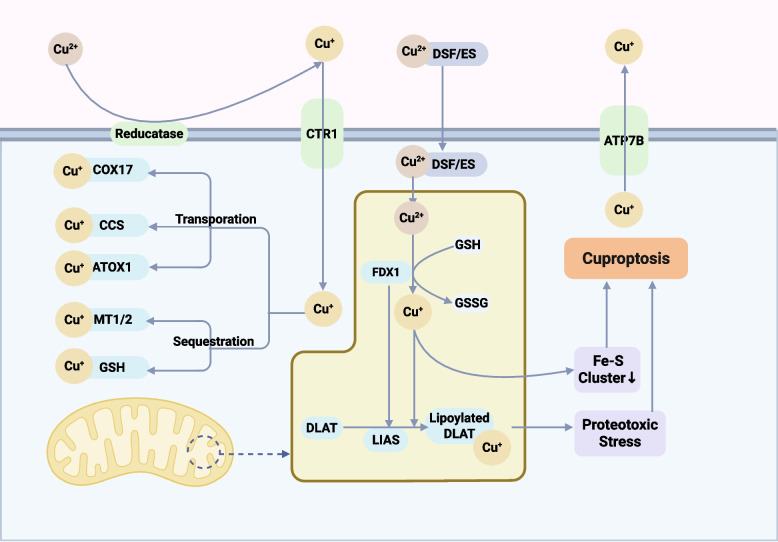
Basic mechanism of cuproptosis. Cuproptosis is a distinct copper-induced cell death characterized by mitochondrial proteotoxic stress. Excess intracellular Cu^2^^+^ (reduced to Cu^+^ ) accumulates in the mitochondrial matrix and binds to lipoylated TCA-cycle enzymes This causes aberrant oligomerization and aggregation of these enzymes, with concurrent loss of Fe–S cluster proteins, ultimately collapsing mitochondrial respiration. The ensuing mitochondrial dysfunction and protein aggregation trigger a unique cell death accompanied by localized oxidative stress. Key regulators of cuproptosis include copper importers and exporters (ATP7A/B) that control cellular Cu levels; the reductase FDX1 which converts Cu^2+^ to toxic Cu^+^ ; and the protein lipoylation pathway that provides the requisite Cu-binding sites. Cells counteract cuproptosis via metallothioneins and heat shock proteins (which sequester copper and refold damaged proteins), but overwhelming copper leads to irreversible proteotoxic stress and cell death. Abbreviations: ATOX1, antioxidant protein 1 (copper chaperone); ATP7A/ATP7B, copper-transporting P-type ATPases; CCS, copper chaperone for superoxide dismutase; COX17, cytochrome c oxidase copper chaperone; CTR1, high-affinity copper transporter 1 (SLC31A1); Cu, copper; DLAT, dihydrolipoamide S-acetyltransferase (PDH-E2); ETC, electron transport chain; FDX1, ferredoxin 1; Fe-S, iron–sulfur; GSH, glutathione; GSSG, oxidized glutathione; LIAS, lipoic acid synthase; LIPT1, lipoyltransferase 1; MT1/2, metallothionein-1/2; MTs, metallothioneins; ROS, reactive oxygen species; TCA, tricarboxylic acid cycle

Cuproptosis is a recently elucidated and unique form of regulated cell death triggered by copper overload, with a core mechanism distinct from known pathways such as apoptosis, ferroptosis, or necroptosis [21, 49, 50]. Unlike apoptosis, cuproptosis is independent of caspase executioners and originates from severe biochemical disturbances within respiring mitochondria [21]. The fundamental cause is that excess copper ions directly target and disrupt key proteins in the tricarboxylic acid (TCA) cycle, triggering a cascade of events that ultimately lead to cell death from unbearable proteotoxic stress and metabolic collapse [22, 51]. This discovery underscores the importance of intracellular metal homeostasis and offers a novel molecular understanding of the biological toxicity of copper [52]. The key steps of this mitochondria-centric cell death pathway are summarized in Fig. 2 of this review.

The molecular mechanism of cuproptosis is centered on the mitochondrion, and its initiation and execution involve a precise and interconnected signaling chain [21, 53]. Intracellular copper concentration is strictly regulated by copper transporters, with SLC31A1 (also known as CTR1) being the primary copper importer. At the same time, ATP7A and ATP7B are responsible for pumping excess copper out of the cell to maintain copper homeostasis [54, 55]. When copper homeostasis is disrupted and a large influx of copper ions enters the cell, the cuproptosis pathway is activated. In this process, ferredoxin 1 (FDX1) plays a crucial upstream regulatory role [56]. As a mitochondrial [2Fe-2S] protein, FDX1 performs a dual key function: it not only efficiently reduces cupric ions (Cu^2^⁺) to the more reactive cuprous ions (Cu⁺) but is also essential for the process of protein lipoylation [52, 56]. Protein lipoylation, the covalent attachment of lipoic acid to lysine residues of specific proteins, is a critical prerequisite for cuproptosis. It generates the direct targets for copper ions, paving the way for the subsequent lethal binding events [57].

The core execution event of cuproptosis is the direct binding of cuprous ions (Cu⁺) to lipoylated protein components of the TCA cycle [21, 22]. The most critical target protein is dihydrolipoamide S-acetyltransferase (DLAT), which is the E2 subunit of the pyruvate dehydrogenase (PDH) complex [21, 58]. Excess Cu⁺ binds to the lipoic acid moieties on DLAT, causing its irreversible oligomerization and aggregation [21, 59]. This protein aggregation directly induces severe mitochondrial dysfunction. First, it induces intense proteotoxic stress, as a large number of misfolded and aggregated proteins accumulate in the mitochondrial matrix, overwhelming the mitochondrion's own processing capacity [51, 60]. Second, this process further induces the loss and degradation of iron-sulfur (Fe-S) cluster proteins [21, 61]. Because Fe-S cluster proteins are essential cofactors for many key enzymes in the mitochondrial electron transport chain (ETC) complexes and the TCA cycle, their loss severely impairs mitochondrial respiration. It blocks ATP production, leading to a cellular energy crisis [41, 62]. Even more fatally, the collapse of the ETC exacerbates electron leakage, leading to an explosive production of ROS and plunging the cell into a state of severe oxidative stress [62]. Therefore, FDX1-mediated copper ion reduction and protein lipoylation serve as the "upstream switch" for cuproptosis, while the aggregation of lipoylated proteins, such as DLAT, the loss of Fe-S cluster proteins, and the ensuing lethal mitochondrial dysfunction act as its "downstream executioner" [53, 63].

### Precision orchestration of shared metabolic hubs​

Although ferroptosis and cuproptosis are two forms of regulated cell death driven by different metal ions and possessing distinct execution mechanisms, they do not exist in isolation. Instead, they are intertwined within the cell through multiple molecular nodes, forming a complex regulatory network [25, 26]. These two pathways exhibit significant crosstalk at the levels of cellular metabolism, organelle functional homeostasis, and upstream signaling [61, 64]. Their interaction is mainly manifested in shared metabolic hubs, a common platform for organellar stress, and overlapping upstream regulatory pathways. These points of intersection make it possible to simultaneously target both death modalities, an approach that is expected to produce a synergistic killing effect [65–67].

The most central and direct node of crosstalk between these two death pathways is the cellular glutathione (GSH)/cysteine metabolic axis [25, 36]. GSH plays a critical inhibitory role in both pathways: it is both the essential cofactor for GPX4 to function and inhibit lipid peroxidation, and an important buffer that chelates excess copper ions to mitigate their toxicity [36, 68]. Therefore, any factor that leads to GSH depletion will simultaneously weaken the cell's resistance to both forms of cell death [36, 69]. Mitochondria serve as the second key platform for ferroptosis–cuproptosis crosstalk, acting as a "reactor" where death signals from both pathways are mutually amplified and transmitted [41, 43, 70]. This crosstalk constitutes a positive feedback loop rather than a simple summation. The core event of cuproptosis is the aggregation of lipoylated proteins, such as DLAT, which directly leads to the stalling of the TCA cycle and the degradation of Fe-S cluster proteins [21]. The degradation of Fe-S cluster proteins, in turn, directly releases their iron ions, expanding the labile iron pool, and causes the collapse of ETC function, leading to massive ROS production and providing a strong oxidative drive for lipid peroxidation [61, 71]. Conversely, the massive lipid peroxidation that occurs during ferroptosis severely damages mitochondrial membrane integrity, leading to mitochondrial dysfunction. This damage makes the mitochondria more sensitive to the toxic effects of copper ions [26].

**Fig. 3:**
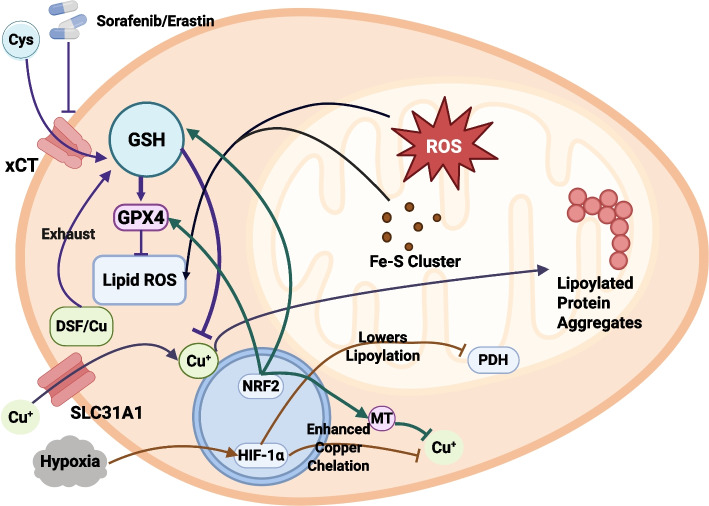
Interplay between ferroptosis and cuproptosis (“double‑strike” strategy). Ferroptosis and cuproptosis are interconnected at multiple levels, and co-inducing them can produce synergistic anti-tumor effects. Glutathione metabolism is a key nexus: for example, disulfiram + Cu depletes GSH, causing lipid ROS accumulation (ferroptosis), while also inducing protein aggregation (cuproptosis); tumor cells respond by upregulating cystine import (System Xc⁻) to replenish GSH under this dual stress. Mitochondrial activity is another common platform – mitochondria-derived ROS amplify ferroptotic lipid peroxidation, and mitochondrial lipoylated enzymes are direct targets of cuproptosis. Thus, mitochondrial dysfunction can modulate susceptibility to both ferroptosis and cuproptosis. Overlapping regulators further link the two: for instance, NRF2 upregulates antioxidant defenses (GSH synthesis, metallothioneins) that buffer both ferroptosis and cuproptosis. Abbreviations: DSF, disulfiram; GPX4, glutathione peroxidase 4; GSH, glutathione; HIF1-α, hypoxia-inducible factor 1-alpha; lipid ROS, lipid reactive oxygen species; MT, mitochondrion/mitochondria (to distinguish from MTs); MTs, metallothioneins; NRF2, nuclear factor erythroid 2–related factor 2; PDH, pyruvate dehydrogenase complex; ROS, reactive oxygen species; SLC31A1, high-affinity copper transporter 1 (CTR1); System Xc⁻, cystine/glutamate antiporter; xCT, SLC7A11 (light chain of System Xc⁻)

These two death pathways also share some upstream master regulatory transcription factors, such as NRF2 and p53, whose activity status can simultaneously determine the cell's sensitivity to both death modalities [72, 73]. NRF2 is the central regulator of the cellular antioxidant stress response [74]. Regarding ferroptosis, NRF2 directly regulates the expression of SLC7A11, GPX4, and ferritin (FTH1). For cuproptosis, NRF2's target genes also include enzymes required for GSH synthesis and metallothioneins (MTs), which can directly chelate copper ions. Thus, NRF2 activation confers dual resistance to both ferroptosis and cuproptosis. In contrast, p53 primarily plays a pro-death role [73]. p53 can promote ferroptosis by inhibiting the transcription of SLC7A11, and the downregulation of SLC7A11 also reduces GSH synthesis, thereby indirectly weakening the cell's defense against cuproptosis [75]. Furthermore, autophagy plays a complex dual role in the crosstalk between these two death pathways [54, 76]. Ferritinophagy is a key upstream initiator of ferroptosis [34], while mitophagy, by clearing damaged mitochondria, can mitigate oxidative stress and thus may inhibit the progression of both ferroptosis and cuproptosis [43]. This complex interplay lays the foundation for a synergistic "dual-strike" strategy, which is illustrated in Fig. 3 of this review.

**Fig. 4:**
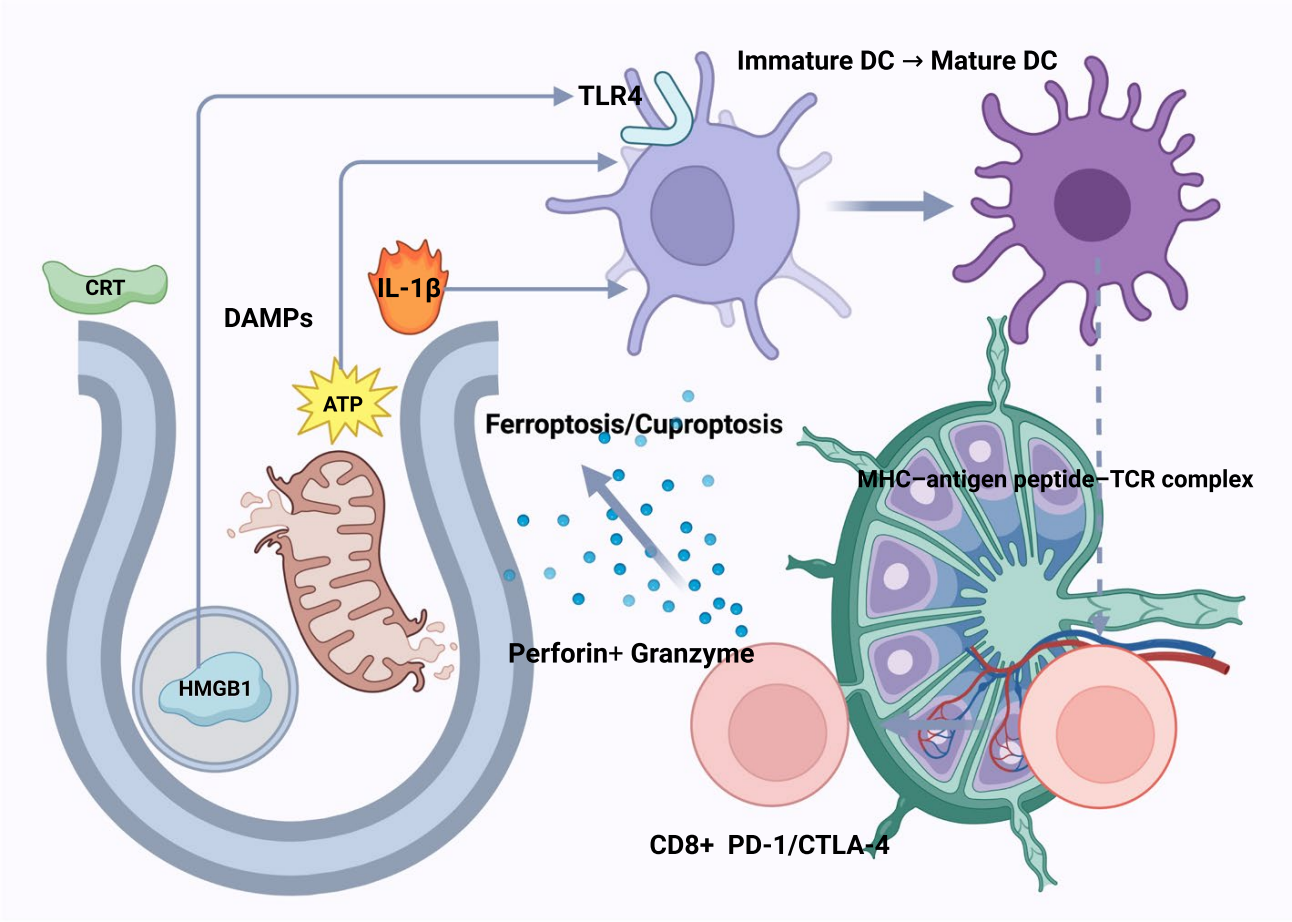
Ferroptosis- and cuproptosis-induced immunogenic cell death and anti-tumor immunity. When tumor cells undergo ferroptosis or cuproptosis, they release danger signals that can provoke an immune response. Ferroptosis, driven by excess lipid peroxidation, causes release of DAMPs and exposure of calreticulin on the cell surface. Cuproptosis likewise leads to DAMP release through protein aggregation-induced stress. These DAMPs act as “find-me” and “eat-me” signals, recruiting and activating dendritic cells (and other antigen-presenting cells) which then prime cytotoxic T cells against tumor antigens. The outcome is an immunogenic cell death cascade in which the immune system recognizes and attacks the remaining tumor cells. Thus, inducing ferroptosis or cuproptosis not only directly kills cancer cells but also heightens tumor immunogenicity – potentially improving immunotherapy efficacy by converting immunologically “cold” tumors into “hot” tumors that respond to treatment. Abbreviations: ATPe, extracellular ATP; CD8, cluster of differentiation 8 (cytotoxic T cell); CRT, calreticulin; CTL, cytotoxic T lymphocyte; CTLA-4, cytotoxic T-lymphocyte–associated protein 4; DAMPs, damage-associated molecular patterns; DC, dendritic cell; HMGB1, high-mobility group box 1; IFN-γ, interferon-gamma; IL-1β, interleukin-1 beta; MHC, major histocompatibility complex; MHC-I, major histocompatibility complex class I; PD-1, programmed cell death protein 1; TAM, tumor-associated macrophage; TCR, T-cell receptor; TLR4, Toll-like receptor 4; TME, tumor microenvironment; Treg, regulatory T cell

Ultimately, a crucial functional intersection of these two death modalities is their ability to induce immunogenic cell death (ICD), thereby converting an intrinsic cellular death signal into an extrinsic anti-tumor immune response [77]. When tumor cells undergo ferroptosis or cuproptosis, they release a series of damage-associated molecular patterns (DAMPs), such as high mobility group box 1 (HMGB1), ATP, and calreticulin (CRT) [78]. These DAMPs act as "danger signals" that can be recognized by the immune system, leading to the recruitment and activation of dendritic cells (DCs), promoting their maturation and antigen presentation [79, 80]. Mature DCs then activate cytotoxic T lymphocytes (CTLs), ultimately triggering a specific immune attack against the tumor [44]. Therefore, inducing ferroptosis or cuproptosis can not only directly kill tumor cells but also, through an "in situ vaccine" effect, transform immunologically "cold" tumors into "hot" tumors infiltrated by immune cells [81]. This process, which links cell death to anti-tumor immunity, is depicted in Fig. 4 of this review. To more clearly distinguish between these death modalities, a comparison of the key features of ferroptosis, cuproptosis, and other relevant RCD pathways is provided in Table 1 [52, 82–84].

**Table 1:** Key features of ferroptosis, cuproptosis, and other metal-mediated RCD pathways

Aspect	Ferroptosis	Cuproptosis	Calcicoptosis
Inducers	Fe^2^⁺ overload; System Xc⁻ inhibition; GPX4 inactivation	Copper overload (Cu^2^⁺ → Cu⁺); Copper ionophores	Ca^2^⁺ overload; mPTP opening; Plasma membrane damage
Core Axis & Key Nodes	System Xc⁻–GSH–GPX4; ACSL4/LPCAT3 (PUFA loading); FSP1–CoQ parallel antioxidation	FDX1; LIAS/LIPT1 (lipoic acid/lipoylation); PDH-E2 (DLAT) aggregation	IP3R/RyR/ORAI1; CypD-mPTP; Calpains
Mitochondrial Role	Not strictly required for initiation, but mitochondrial ROS and Fe-S homeostasis can amplify lipid peroxidation	Strictly required (protein aggregation in matrix, Fe-S protein loss, ETC collapse)	Central event (depolarization, swelling, rupture via mPTP)
Hallmark Event	Iron-dependent lipid peroxidation	Proteotoxic aggregation of lipoylated TCA cycle enzymes	Mitochondrial permeability transition
Biomarkers/Readouts	MDA/4-HNE ↑; Labile Fe^2^⁺ ↑; PTGS2 transcription ↑; Shrunken mitochondria	Insoluble aggregates of DLAT ↑; Fe-S proteins ↓; FDX1/LIAS levels	Sustained cytosolic Ca^2^⁺ ↑; ΔΨm loss; Calpain substrate cleavage
Representative Agents	Inducers: Erastin, RSL3, Sorafenib Inhibitors: Ferrostatin-1, Liproxstatin-1, DFO	Inducers: Elesclomol-Cu, Disulfiram → CuET Inhibitors: TM	Inducers: A23187, Ionomycin Inhibitors: BAPTA-AM, Cyclosporin A

*Abbreviations*: *mPTP* mitochondrial permeability transition pore, *DFO* Deferoxamine, *TM* Tetrathiomolybdate

## Metabolic vulnerabilities in GI cancers

A deep understanding of these core molecular mechanisms is the prerequisite for identifying and exploiting the unique metabolic vulnerabilities that GI tumors develop to adapt to malignant proliferation.

### Shared metabolic reprogramming creates exploitable vulnerabilities

To meet the demands of uncontrolled proliferation and adapt to the harsh tumor microenvironment, gastrointestinal (GI) tumor cells systematically remodel their metabolic networks [23, 85]. These metabolic adaptations, mediated by oncogenic signaling or microenvironmental pressures, confer a survival advantage but also create therapeutically exploitable 'metabolic vulnerabilities' [29, 86]. A dependency on specific metabolic pathways characterizes such vulnerabilities. Disruption of these pathways compromises cellular homeostasis and sensitizes cells to regulated cell death, including ferroptosis or cuproptosis [17]. This section will conceptually elaborate on the common vulnerability axes in GI tumors related to lipid, amino acid, metal ion, and energy metabolism, as well as their antioxidant defense systems, laying the theoretical groundwork for the subsequent in-depth discussion of specific molecular mechanisms in each cancer type.

#### Lipid reprogramming and peroxidation risk

The lipid composition of the cell membrane is the physical basis that determines sensitivity to ferroptosis [31]. Compared to normal cells, highly proliferative GI tumor cells must synthesize large quantities of biomembranes, which forces them to reprogram their lipid metabolism pathways. This reprogramming manifests as two antagonistic yet balanced aspects, creating a fragile equilibrium. On the one hand, to avoid lipid peroxidation induced by high metabolic activity and oxidative stress, tumor cells generally upregulate the synthesis of monounsaturated fatty acids (MUFAs), a process primarily catalyzed by stearoyl-CoA desaturase 1 (SCD1) [74]. Although this adaptive change enhances their resistance to ferroptosis, it also makes them highly dependent on SCD1 activity. If SCD1 is inhibited, the cells cannot handle the toxicity of saturated fatty acids and face a crisis in membrane fluidity. On the other hand, the incorporation of polyunsaturated fatty acids (PUFAs) into cell membranes is essential for the execution of ferroptosis [32]. Therefore, changes in the expression and activity of key enzymes regulating PUFA metabolism, such as ACSL4 and LPCAT3, directly affect the "flammability" of tumor cells. The conflicting demand between the "pro-survival" synthesis of MUFAs and the "pro-death" incorporation of PUFAs makes lipid metabolism a common metabolic vulnerability in GI tumors.

#### Amino acid addiction and redox defense

The persistently high levels of reactive oxygen species (ROS) in GI tumor cells make them highly dependent on the GSH-centered antioxidant system, and GSH synthesis is entirely reliant on the supply of specific amino acids. This constitutes the vulnerability of "amino acid addiction" [23]. Compared to most normal tissues, the demand for cystine/cysteine in tumor cells is dramatically increased, leading them to frequently overexpress the cystine/glutamate antiporter System Xc⁻ to ensure an adequate GSH pool [35]. This over-reliance on a single transporter (SLC7A11) provides a clear therapeutic window, as inhibiting it has a far more negligible impact on normal cells than on tumor cells. Similarly, the "craving" of tumor cells for glutamine is not only for replenishing the tricarboxylic acid cycle but also for obtaining the glutamate backbone required for GSH synthesis and for producing NADPH to neutralize ROS [74]. Therefore, by restricting the supply of specific amino acids or inhibiting their metabolic pathways, it is possible to directly attack the core redox defense system of GI tumors, exposing their survival defects under high oxidative stress.

#### Dysregulated metal ion homeostasis

Iron and copper are essential cofactors for hundreds of intracellular enzymes and are crucial for DNA replication, energy metabolism, and signal transduction [17]. To meet the demands of their accelerated proliferation rate, GI tumor cells must upregulate the uptake and utilization of these metal ions [60]. However, these metal ions themselves are catalytically active in redox reactions, and their intracellular concentrations must be precisely controlled to avoid severe toxicity. This contradictory state of both "craving" and "fearing" metal ions makes their homeostatic regulation an easily disrupted equilibrium. Tumor cells increase iron uptake by upregulating the transferrin receptor, but they must also enhance their iron storage (ferritin) and export capabilities to prevent the excessive expansion of the labile iron pool, which would trigger Fenton reactions and ferroptosis [34]. Similarly, while a dependency on copper (cuproplasia) can promote angiogenesis, it also exposes cells to the risk of copper overload. Once the buffering capacity is exceeded, cuproptosis will be triggered within the mitochondria [87]. Thus, although the adaptive remodeling of metal ion homeostasis in GI tumors supports their malignant phenotype, it also makes them exceptionally sensitive to external interventions that can further disrupt this fragile balance.

#### Mitochondrial integrity and quality control

Although the "Warburg effect" is a hallmark of energy metabolism in GI tumors, this does not imply a loss of mitochondrial function, but rather its remodeling [23]. Tumor cells still rely on mitochondria to perform essential biosynthetic tasks, and the mitochondrial respiratory chain is a major source of endogenous ROS [41]. This high-load operational state places the mitochondria of tumor cells on the verge of damage and stress, making the cells highly dependent on mitochondrial quality control (MQC) mechanisms, such as mitophagy, to clear dysfunctional organelles [43]. This dependency on MQC is itself a vulnerability. The execution of cuproptosis is entirely dependent on functional mitochondria as its target, while the occurrence of ferroptosis is significantly amplified by mitochondria-derived ROS [21, 29]. Therefore, any intervention that can interfere with the MQC pathways of tumor cells or exacerbate mitochondrial stress has the potential to breach the cell's tolerance threshold and synergistically induce ferroptosis or cuproptosis.

#### Dependence on antioxidant systems

To survive in a microenvironment of high oxidative stress, GI tumor cells universally activate their endogenous antioxidant defense systems [88]. The transcriptional program centered on the KEAP1-NRF2 signaling axis is one of their most critical survival mechanisms [74]. In many GI tumors, the NRF2 pathway is constitutively activated due to oncogenic mutations or persistent oxidative stress, leading to the overexpression of numerous antioxidant genes (including GSH synthesis enzymes, the thioredoxin system, and GPX4), thereby building a powerful anti-ferroptotic barrier [89]. However, this state also makes the tumor cells "addicted" to the continuous activation of the NRF2 pathway. Unlike normal cells, which can flexibly adjust their antioxidant levels, these tumor cells have set their survival baseline at a state requiring high levels of NRF2 activity. Consequently, the NRF2 pathway itself, as well as its key downstream effectors (like GPX4), transforms from a protective factor into a vulnerability that, if inhibited, leads to cellular collapse, providing clear targets for therapeutic intervention.

### Distinct regulatory networks govern cell death sensitivity in GI cancers

The clinical application of therapies targeting ferroptosis and cuproptosis requires a context-specific understanding that extends beyond common metabolic vulnerabilities. The efficacy of inducing these death pathways is determined by the distinct molecular landscapes, oncogenic drivers, and tumor microenvironments of each GI cancer type. This section outlines the specific regulatory networks of ferroptosis and cuproptosis in major gastrointestinal malignancies, aiming to identify disease-specific pathways and therapeutic targets. The anatomical sequence of the gastrointestinal tract organizes the content.

#### Gastric Cancer (GC)

In gastric cancer (GC), the regulatory networks of ferroptosis and cuproptosis are particular, centering on the precise control of core effector molecules by key transcription factors, signaling pathways, and post-translational modification systems. These mechanisms collectively shape the sensitivity of GC cells to metal-dependent cell death [90–92]. Oncogene-driven transcriptional reprogramming is tightly coupled with metabolic adaptations, forming the basis for GC to evade these cell death pathways [90, 93].

A multi-layered, multi-pathway inhibitory network characterizes the regulation of ferroptosis in GC. At the transcriptional level, the lineage-survival transcription factors GATA4 and GATA6 have been shown to regulate the expression of genes associated with multiple cellular phenotypes, including ferroptosis [90]. Similarly, the transcription factor YY1 inhibits ferroptosis in GC cells by modulating the p53 signaling pathway, thereby mediating resistance to apatinib [91]. The convergence of multiple distinct oncogenic transcription factors on ferroptosis inhibition indicates that evading this cell death pathway is a critical hurdle for GC progression. At the signal transduction level, the classic PI3K/AKT pathway plays a key role through the downstream AKT/GSK3β/NRF2 axis [93]. In this pathway, AKT activation inhibits GSK3β, which in turn relieves the inhibition of NRF2, resulting in its stabilization and subsequent activation. This ultimately upregulates the expression of its downstream target gene, GPX4, thereby establishing a formidable anti-ferroptotic defense [93]. Furthermore, autophagy plays a crucial role in regulating ferroptosis. On one hand, NCOA4-mediated ferritinophagy is a key upstream event that induces ferroptosis by degrading ferritin (FTH1) and expanding the labile iron pool [94]. On the other hand, NPR1 promotes cisplatin resistance in GC by inhibiting PARL-mediated, mitophagy-dependent ferroptosis, which prevents the release of iron from damaged mitochondria [95]. The opposing functions of NCOA4 (pro-ferroptotic) and NPR1 (anti-ferroptotic) reveal a dynamic equilibrium within GC cells that determines their susceptibility to ferroptosis through the precise regulation of autophagy.

The regulatory mechanisms of cuproptosis in GC are also precise, particularly at the levels of RNA processing and post-translational modification. A key study found that the RNA-binding protein PTBP3 is highly expressed in peritoneal metastatic GC. It mediates exon four skipping in the pre-mRNA of its target gene, COX11, producing a functionally impaired short transcript [96]. The inactivated COX11 protein cannot effectively maintain mitochondrial copper homeostasis, resulting in reduced intracellular copper levels and enabling tumor cells to evade cuproptosis [96]. This finding directly links aberrant RNA splicing, a common feature of cancer, to resistance against a specific form of cell death, revealing a new role for the "splicing code" in regulating metabolic vulnerability. Additionally, post-translational modifications of proteins play a role in the fine-tuning of cuproptosis. Under copper stress, methyltransferase-like 16 (METTL16) undergoes lactylation at lysine 229 (K229). This modification enhances the activity of the m6A methyltransferase, specifically catalyzing the m6A modification of the mRNA of the core cuproptosis factor FDX1, thereby regulating its expression and executing the cuproptosis program [92]. This discovery for the first time directly links the cellular metabolic state (lactylation) and RNA epigenetics (m6A) to the execution of cuproptosis. It provides a novel rationale for developing drugs that target this "metabolic-epigenetic" axis to sensitize cells to cuproptosis-inducing therapies.

In GC, mechanistic crosstalk exists between ferroptosis and cuproptosis. The integrin subunit ITGAV is involved in regulating both cell death pathways [97]. As a key molecule connecting the extracellular matrix (ECM) to intracellular signaling, the dual regulatory role of ITGAV suggests that signals from the tumor microenvironment may act as an upstream master switch, coordinating the cellular response to metabolic stress induced by two different metal ions, iron and copper, thus forming a higher-level regulatory network.

#### Colorectal Cancer (CRC)

The regulatory networks of ferroptosis and cuproptosis in colorectal cancer (CRC) are profoundly influenced by its unique molecular background, particularly the mutation status of key oncogenes, such as KRAS and BRAF, as well as the direct involvement of the gut microbiome [98, 99]. These factors collectively constitute a complex regulatory system that determines the sensitivity of CRC cells to these two cell death modalities.

Oncogenic signaling pathways play a central role in CRC's evasion of ferroptosis. In BRAF V600E-mutant CRC, adaptive resistance to BRAF/EGFR inhibitors is achieved through the upregulation of GPX4, with the upstream mechanism involving the Polo-like kinase 1 (PLK1) signaling axis [98]. Activated PLK1 phosphorylates the chromobox protein homolog 8 (CBX8), which in turn drives the transcription of GPX4, establishing a robust anti-ferroptotic defense [98]. Furthermore, the Wnt signaling pathway transcriptionally upregulates SLC7A11 through its receptor LGR4, thereby inhibiting ferroptosis and contributing to drug resistance [100]. The convergence of these parallel signaling pathways on the inhibition of ferroptosis highlights it as a critical node in CRC therapeutic resistance. The gut microbiome, as a unique external regulatory layer in CRC, directly influences the ferroptosis process. For instance, indole-3-propionic acid (IDA), a metabolite from *Peptostreptococcus anaerobius*, activates the aryl hydrocarbon receptor (AHR) signaling pathway, which upregulates ALDH1A3 and ultimately enhances the FSP1-dependent CoQ10 synthesis pathway, thereby effectively inhibiting ferroptosis [99]. Conversely, γ-linolenic acid (γ-LA) produced by *Lactobacillus plantarum* promotes ferroptosis by inducing mitochondrial damage [101]. Collectively, these findings indicate that the ferroptotic fate of CRC cells is determined by a dynamic interplay between intrinsic oncogenic signals and opposing 'pro-death' and 'anti-death' metabolites derived from the gut microenvironment. This interaction provides a direct theoretical basis for remodeling tumor ferroptosis sensitivity by modulating the microbiome.

Post-translational modifications act as molecular switches regulating ferroptosis in CRC. Chemotherapy-activated MAPK signaling leads to ERK-mediated phosphorylation of the RFNG protein. Phosphorylated RFNG translocates to the nucleus, inhibits p53 function, and ultimately upregulates SLC7A11 expression, thereby inhibiting ferroptosis and constituting a complex adaptive resistance mechanism [102]. In addition, lactylation of histone deacetylase 1 (HDAC1) at lysine 412 directly links the cell's metabolic state (lactate levels) to the epigenetic regulation of ferroptosis-related genes. High lactate levels inhibit ferroptosis through this modification, thereby connecting the Warburg effect with ferroptosis evasion [103]. Another key regulatory mechanism of ferroptosis involves the ATF3-CBS signaling axis, which is activated under cysteine deprivation stress and enhances the resistance of CRC cells to ferroptosis by targeting the mitochondrial tricarboxylic acid cycle [104].

The regulation of cuproptosis in CRC is also finely controlled by epigenetics and non-coding RNAs. The long non-coding RNA (lncRNA) PVT1 acts as a molecular scaffold, recruiting the DDX5/p300/MYB complex to the promoter region of the core cuproptosis gene FDX1 [105]. The p300, a histone acetyltransferase in this complex, catalyzes the acetylation of H3K27 in the promoter region, thereby opening the chromatin structure, activating FDX1 transcription, and ultimately enhancing the cell's sensitivity to cuproptosis [105]. Apparent mechanistic crosstalk exists between ferroptosis and cuproptosis in CRC, with glutathione (GSH) as the core node. GSH is not only the necessary substrate for GPX4 function (inhibiting ferroptosis) but also an effective copper ion chelator (inhibiting cuproptosis) [106]. Therefore, when cells are overloaded with copper ions, GSH is heavily consumed. This depletion of a shared resource not only directly promotes cuproptosis but also causes the GPX4 pathway to fail due to substrate deficiency, thereby synergistically triggering ferroptosis [106]. This hierarchical relationship, where copper overload unilaterally triggers ferroptosis, positions GSH as the central molecule in the crosstalk between the two death pathways.

#### Hepatocellular Carcinoma (HCC)

The molecular pathology of hepatocellular carcinoma (HCC), characterized by viral infection, chronic inflammation, and complex genomic alterations, creates a metabolic phenotype sensitive to iron and copper dyshomeostasis [107]. The regulatory networks for ferroptosis and cuproptosis in HCC are controlled by deubiquitinating enzymes (DUBs), RNA epigenetic modifications, and specific reprogramming of trace element metabolism, such as selenium [108, 109].

HCC cells have evolved a highly robust ferroptosis defense system composed of multiple DUBs and epigenetic factors. For instance, USP14 enhances radioresistance by deubiquitinating and stabilizing GPX4 [110]; USP8 promotes the O-GlcNAcylation of SLC7A11 by stabilizing O-GlcNAc transferase (OGT), thereby enhancing its function and inhibiting ferroptosis [109]; and USP18 leads to sorafenib resistance by de-ISGylating NCOA4 and mediating its degradation, which inhibits ferritinophagy and ferroptosis [111]. At the RNA epigenetic level, the methyltransferase METTL3 stabilizes the mRNA of SLC7A11 through m6A modification, thereby inhibiting ferroptosis and promoting radioresistance [112]. This multi-level, redundant regulatory network targeting different components of the ferroptosis pathway (GPX4, SLC7A11, NCOA4) highlights the extreme importance of inhibiting ferroptosis for the survival of HCC.

The regulation of cuproptosis in HCC is also closely related to protein stability and cellular metabolic state. The deubiquitinase UCHL3 stabilizes pyruvate kinase M2 (PKM2) by removing its ubiquitin chains, thereby enhancing the activity of the glycolysis pathway [113]. Since the product of PKM2 catalysis, pyruvate, is a key substrate for the TCA cycle, and the lipoylated proteins within the TCA cycle (like DLAT) are the direct targets of cuproptosis, the stabilizing effect of UCHL3 on PKM2 directly links the cell's glycolytic state to its sensitivity to cuproptosis [113]. Conversely, maternal embryonic leucine zipper kinase (MELK) promotes HCC progression by activating the PI3K/mTOR signaling pathway, which promotes the expression of DLAT and thereby inhibits cuproptosis [114].

HCC exhibits a unique vulnerability to selenium metabolism. In HCC cells, NRF2 activation transcriptionally represses the expression of selenoprotein P (SELENOP), the main protein responsible for transporting selenium out of the cell [108]. The downregulation of SELENOP leads to the retention of intracellular selenium, and this "hoarded" selenium is preferentially used for the synthesis of the critical anti-ferroptotic selenoprotein, GPX4, thereby greatly enhancing the ability of HCC cells to resist ferroptosis [108]. This "selfish" mechanism, by which tumor cells hijack systemic nutrient allocation to enhance their own survival, constitutes a specific metabolic reprogramming strategy in HCC.

In HCC, direct mechanistic crosstalk exists between ferroptosis and cuproptosis, with GSH as the core hub molecule [115]. Ferroptosis inducers, such as sorafenib, inhibit the function of System Xc⁻, leading to impaired intracellular GSH synthesis [116]. The depletion of GSH has a dual effect: on one hand, GPX4 is inactivated due to a lack of substrate, directly triggering ferroptosis; on the other hand, the reduction of GSH, the cell's main copper ion chelator, makes the cell extremely sensitive to copper toxicity, thereby synergistically promoting cuproptosis [115]. Furthermore, master regulators like p53 and NRF2 have also been shown to simultaneously regulate both pathways, indicating that the cellular response to specific metabolic stresses may be coordinately regulated through these central signaling hubs [115].

#### Pancreatic Ductal Adenocarcinoma (PDAC)

Pancreatic ductal adenocarcinoma (PDAC) exhibits extreme therapeutic resistance and unique KRAS-driven metabolic reprogramming, which affects its response to ferroptosis and cuproptosis [117, 118]. PDAC cells utilize complex signaling and metabolic networks to suppress these cell death pathways, thereby ensuring their survival.

KRAS mutation is the central driving event in PDAC and confers ferroptosis resistance through multiple pathways. On the one hand, KRAS upregulates the expression of the actin-regulatory protein TMOD3 through its downstream ETS transcription factor, ELK1 [119]. TMOD3 promotes F-actin polymerization, which accelerates the fusion of autophagosomes with lysosomes, thereby enhancing the autophagic degradation of the key positive ferroptosis regulator ACSL4 and ultimately inhibiting ferroptosis [119]. On the other hand, the KRAS signaling pathway, particularly the activation of NRF2, is a key mechanism of ferroptosis resistance in PDAC [117]. Post-translational modifications also play a critical role in regulating ferroptosis in PDAC. Salt-inducible kinase 1 (SIK1) protects HDAC5 from TRIM28-mediated ubiquitination and degradation by phosphorylating it at Ser498 [120]. Stabilized HDAC5 then deacetylates STAT6 and enhances its transcriptional activity, thereby driving the expression of SLC7A11 and conferring ferroptosis resistance to PDAC cells [120]. This multi-level modification cascade involving phosphorylation, ubiquitination, and deacetylation elegantly links cellular energy sensing (SIK kinase), epigenetic regulation (HDAC5), and signal transduction (STAT6) to jointly control the gate of ferroptosis. This complex resistance network, dominated by the core driver gene (KRAS) and involving protein stability, autophagy regulation, and multi-level signaling modifications, explains why single-target ferroptosis inducers are often of limited efficacy in PDAC, highlighting the necessity of developing combination therapeutic strategies that can dismantle the entire resistance network.

The metabolic vulnerability of PDAC is closely related to its dependence on specific nutrients and the reprogramming of lipid metabolism. Acyl-CoA thioesterase 8 (ACOT8) confers resistance to gemcitabine. It inhibits ferroptosis by regulating intracellular cholesteryl ester (CE) levels and reducing the levels of phosphatidylethanolamines (PEs) containing polyunsaturated fatty acids, thereby inhibiting peroxisome activation and lipid peroxidation [118]. Additionally, the dependence of PDAC cells on glutamine constitutes an exploitable weakness, as glutamine deprivation can induce the initiation of the ferroptosis program [121].

In PDAC, profound and direct mechanistic crosstalk exists between ferroptosis and cuproptosis. A key finding is that copper ions can directly induce the autophagic degradation of GPX4, thereby triggering ferroptosis [122]. In this mechanism, intracellular copper overload leads to the aggregation of GPX4, which is then recognized by the autophagy receptor TAX1BP1 and subsequently degraded via the autophagy pathway [122]. This finding positions copper as a potent upstream regulator of ferroptosis, revealing that copper ions can not only induce cuproptosis by targeting lipoylated proteins of the TCA cycle but can also initiate the ferroptosis program by directly destroying the core anti-ferroptotic enzyme GPX4. Furthermore, GSH plays a dual inhibitory role in PDAC, inhibiting cuproptosis by chelating copper ions and inhibiting ferroptosis as a substrate for GPX4, thereby serving as a key metabolic node that connects the two pathways [123]. Regarding cuproptosis regulation, the key EMT transcription factor TWIST1 promotes glycolysis and cuproptosis by stabilizing hexokinase 2 (HK2) protein levels through the inhibition of its ubiquitination-mediated degradation [124].

#### Biliary Tract Cancers (BTC)

The development and progression of biliary tract cancers (BTC), including cholangiocarcinoma (CCA) and gallbladder cancer (GBC), involve the dysregulation of the ferroptosis regulatory network [125, 126]. As the primary organelle for ferroptosis execution, the mitochondrion integrates multiple signaling pathways in BTC, and its functional integrity is a key determinant of tumor cell fate [127, 128].

Core regulators of ferroptosis play a key role in BTC. The expression levels of ACSL4 and SLC7A11 are important biomarkers for determining the ferroptosis sensitivity of CCA cells, with high expression often associated with tumor progression and poor prognosis [125]. In GBC, DNA methylation in the promoter region of the tumor suppressor RUNX3 is a key mechanism leading to its silencing [126]. Inactivated RUNX3 cannot effectively transactivate its downstream targets, leading to the suppression of SLC7A11-mediated ferroptosis and promoting malignant tumor progression [126]. This strategy of chronically shutting down a pro-ferroptotic pathway through stable epigenetic modification highlights the intense selective pressure for BTC to evade ferroptosis for survival.

Multiple signaling axes ultimately converge on the mitochondrion to jointly regulate the ferroptosis process in BTC. The mitochondrial protein FUNDC1 promotes CCA progression by inhibiting ferroptosis through its interaction with the GTPase RAC1 [127]. Another key axis is the ROCK2/UBA52/Drp1 pathway, which confers resistance to the FGFR inhibitor pemigatinib in CCA by stabilizing the mitochondrial fission protein Drp1, inhibiting its ubiquitination-mediated degradation, and thereby altering mitochondrial dynamics to suppress ferroptosis [128]. Furthermore, signals from the gut microenvironment can also influence the ferroptosis sensitivity of BTC. In intrahepatic cholangiocarcinoma (ICC), the gut microbiota can regulate the ALK5/NOX1 signaling axis by altering host glutamine metabolism, ultimately inhibiting ferroptosis in tumor cells [129]. These findings collectively point to the mitochondrion as the central platform in BTC for integrating various intrinsic and extrinsic signals to determine the fate of ferroptosis. This research gap may reflect the relatively low incidence of BTC or the lack of research models; however, given that bile is naturally rich in copper ions, this highlights a future research direction with great potential: systematically exploring the role of cuproptosis in the development of BTC may reveal its unique therapeutic vulnerabilities.

#### Esophageal Cancer (ESCC/EAC)

A significant obstacle in the treatment of esophageal cancer, particularly esophageal squamous cell carcinoma (ESCC) and esophageal adenocarcinoma (EAC), is radioresistance. Ferroptosis and cuproptosis, as emerging cell death modalities, have been confirmed to be key mechanisms that determine and even mediate the response to radiotherapy [130, 131].

ESCC cells employ multiple mechanisms to inhibit ferroptosis, thereby enhancing their survival and resistance to therapy. At the level of ion transport, the zinc transporter ZIP8 is highly expressed in ESCC. It increases intracellular zinc ion concentration, which promotes the phosphorylation of CREB, leading to the transcriptional upregulation of GPX4 and ultimately inhibiting ferroptosis [132]. This discovery reveals a significant crosstalk between zinc ion homeostasis and iron-dependent cell death. At the metabolic enzyme level, both acetyl-CoA acetyltransferase 2 (ACAT2) and the aldo–keto reductase AKR1C3 have been shown to confer radioresistance to ESCC and esophageal adenocarcinoma (EAC) by inhibiting ferroptosis [130, 133]. At the post-transcriptional level, ESCC cells finely regulate both major branches of the ferroptosis defense system: the GPX4 enzyme itself and the substrate supply system (System Xc⁻). The long non-coding RNA TMEM44-AS1 stabilizes GPX4 mRNA by recruiting the RNA-binding protein IGF2BP2, thereby maintaining high levels of GPX4 protein [134]. Concurrently, the downregulation of the E3 ubiquitin ligase NEDD4L reduces the ubiquitination-mediated degradation of the core System Xc⁻ subunit xCT (SLC7A11), thus ensuring a continuous influx of cystine and synthesis of GSH [135]. This dual regulatory strategy, targeting both the enzyme and its substrate supply, constructs a robust anti-ferroptotic defense in ESCC.

Cuproptosis has been revealed as a direct effector mechanism of cell death induced by radiotherapy (RT) in ESCC, a process that is independent of ferroptosis and apoptosis [131]. RT triggers cuproptosis by upregulating the expression of the copper transporter CTR1, while simultaneously depleting the mitochondrial copper chelator GSH, resulting in a sharp increase in mitochondrial copper ion levels [131]. ESCC cells can acquire radioresistance by downregulating the transcription factor BACH1, as its downregulation relieves the repression of its target genes encoding the copper-chelating metallothioneins (MTs). High levels of MTs can effectively buffer the RT-induced copper overload [131]. Additionally, PLEKHA7 has also been identified as a key protein that inhibits cuproptosis and promotes radioresistance [136]. Although the core execution mechanisms of ferroptosis and cuproptosis differ, in the context of RT, they can be co-activated by an common upstream stress signal, which is the large amount of ROS generated by ionizing radiation. ROS can both initiate lipid peroxidation (triggering ferroptosis) and deplete mitochondrial GSH (creating the conditions for cuproptosis). Therefore, RT can be considered a "master stressor" that simultaneously attacks multiple metabolic vulnerabilities, leading to the synergistic activation of both death pathways.

In summary, a cross-cancer analysis of GI malignancies reveals distinct patterns of resistance. Ferroptosis resistance is frequently acquired via antioxidant reprogramming, involving key players such as NRF2, GPX4, and System Xc⁻. In contrast, the modulation of cuproptosis sensitivity often involves mechanisms such as RNA processing, epigenetic regulation, and control of mitochondrial protein stability. This divergence demonstrates that while these tumors share a fundamental reliance on metabolic remodeling for survival, the specific molecular nodes they exploit are often cancer-type–specific, thereby presenting both common vulnerabilities and unique therapeutic opportunities.

## Therapeutic intervention strategies

An understanding of the regulatory mechanisms and metabolic vulnerabilities of ferroptosis and cuproptosis in GI tumors has led to the development of various therapeutic strategies designed to activate these pathways. The approaches include small-molecule drugs, nanotechnology platforms, and synergistic combinations with established therapies.

### Small molecules serve as foundational tools for pathway modulation

Small-molecule compounds are the cornerstone tools for exploring and inducing ferroptosis and cuproptosis. They precisely regulate the initiation and execution of cell death by targeting key proteins in the core pathways.

The induction of ferroptosis is primarily achieved through two classic routes: inhibiting the cystine/glutamate antiporter System Xc⁻ or directly inhibiting glutathione peroxidase 4 (GPX4) [137]. Erastin, the first discovered ferroptosis inducer, blocks the function of System Xc⁻, leading to impaired cellular cystine uptake, which in turn depletes glutathione (GSH). This ultimately inactivates GPX4, causing the accumulation of lipid peroxides [36, 138]. Some clinically approved drugs have also been shown to exhibit a similar mechanism of action. For example, the anti-inflammatory drug sulfasalazine (SSZ) effectively inhibits System Xc⁻. In colorectal cancer (CRC) models, it not only induces ferroptosis but also enhances the cytotoxicity of cisplatin by depleting GSH [35]. As a clinically long-used anti-inflammatory drug, the favorable safety profile of sulfasalazine makes it a promising "old drug, new use" candidate. Clinical trials are currently evaluating its application in cancer therapy. Another class of ferroptosis inducers directly targets GPX4, with RSL3 (RAS-selective lethal 3) being the most representative. It covalently binds to the active site of GPX4, directly inactivating it and thereby efficiently inducing ferroptosis [139]. Furthermore, numerous natural products have been discovered to possess ferroptosis-inducing potential. For instance, β-Lapachone induces ferroptosis in CRC cells by activating the JNK pathway, which promotes NCOA4-mediated ferritinophagy and increases the intracellular labile iron pool [140]. The metabolite γ-linolenic acid from *Lactobacillus plantarum* triggers ferroptosis in CRC cells by damaging mitochondria [101]. A more detailed summary of these small-molecule compounds, which regulate cell death by targeting core pathways, along with their mechanisms and applications in gastrointestinal cancers, is provided in Table 2.

**Table 2:** Small-molecule interventions targeting ferroptosis and cuproptosis

Category	Compound/Agent	Mechanism/Target	Applications in GI Cancers	Ref
Ferroptosis Inducers				
	*System Xc⁻ Inhibitors*			
	Erastin	Inhibits System Xc⁻ (SLC7A11), blocks cystine uptake, depletes GSH	CRC, PDAC (classic ferroptosis inducer, used in numerous preclinical models)	[141]
	Sulfasalazine (SAS)	System Xc⁻ inhibitor; blocks cystine uptake and depletes GSH to induce ferroptosis	CRC (inhibits growth and sensitizes to cisplatin and radiotherapy)	[35]
	IKE (Imidazole Ketone Erastin)	Analog of erastin; potent inhibitor of System Xc⁻	Multiple GI cancers (used in preclinical models to induce ferroptosis)	[68]
	*GPX4 Inhibitors/Degraders*			
	RSL3	Covalently inhibits GPX4 peroxidase activity, causing uncontrolled lipid peroxidation	Multiple GI cancer cell lines (potent experimental tool for direct GPX4 inhibition)	[19]
	FIN56	Induces ferroptosis by degrading GPX4 protein and reducing CoQ10 levels	Multiple cancer types (tool compound representing a specific GPX4 degradation mechanism)	[142]
	*Other Mechanisms*			
	Sorafenib	Multikinase inhibitor; Inhibits System Xc⁻ (SLC7A11), depletes GSH, induces ferroptosis	HCC (induces ferroptosis; its efficacy is modulated by various signaling pathways)	[143]
	Baicalein	Induces ferroptosis by blocking the JAK2/STAT3/GPX4 axis	CRC (inhibits tumor growth in xenograft models and shows potential to overcome chemotherapy resistance)	[144]
	FINO2	Endoperoxide-containing compound; Induces ferroptosis by oxidizing iron and indirectly inhibiting GPX4	Multiple cancer types (serves as a tool compound for a distinct mechanism of induction)	[145]
	Brequinar	DHODH inhibitor; blocks a mitochondrial anti-ferroptotic pathway	Multiple cancer models (selectively suppresses GPX4-low tumor growth by inducing ferroptosis)	[48]
	APR-246 (Eprenetapopt)	Initially known as a p53 reactivator; now shown to deplete GSH and induce ferroptosis	Esophageal Cancer, AML (synergizes with serine/glycine restriction)	[46]
Cuproptosis Inducers				
	Elesclomol (in presence of Cu)	Copper ionophore; increases mitochondrial Cu⁺, causing lipoylated protein aggregation and cuproptosis	HCC, CRC (potent cuproptosis inducer targeting mitochondrial metabolism)	[59]
Dual-Inducers/Synergistic Agents				
	Disulfiram (in presence of Cu)	Forms CuET complex, a copper ionophore; induces proteotoxic stress and cuproptosis	HCC, multiple GI tumors (repurposed drug, induces cuproptosis by targeting NPL4)	[36]
Natural Products with Modulating Effects				
	Periplocymarin (PPM)	Cardiac glycoside; Upregulates TFRC via YAP/TAZ, increases iron uptake and lipid peroxidation	GC (sensitizes tumors to cisplatin-induced ferroptosis)	[146]
	Vorinostat (SAHA)	Histone deacetylase (HDAC) inhibitor; induces lipid peroxidation and ferroptosis	GC (enhances chemosensitivity by triggering ferroptosis)	[147]
	Icariin	Natural flavonoid; Induces ferroptosis and enhances anti-tumor immunity	CRC (induces ferroptosis and synergizes with anti-PD-1 immunotherapy)	[148]
	Quercetin	Natural flavonoid; Modulates multiple cell death pathways including ferroptosis and cuproptosis	Multiple GI cancers (preclinical studies show induction of apoptosis and autophagy)	[149]
	Curcumin	Natural polyphenol; Modulates metal ion homeostasis and induces ferroptosis/cuproptosis	HCC (demonstrates cell-specific effects on ferroptosis and cuproptosis)	[150]
	Artemisinin & derivatives	Sesquiterpene lactone; induce ferroptosis through iron-dependent mechanisms	PDAC, multiple GI cancers (preclinical studies show potent anti-tumor activity)	[151]
	Withaferin A	Natural steroidal lactone; Induces ferroptosis by targeting annexin A2	Multiple cancer models (induces ferroptosis in various cancer cell lines)	[152]
	Parthenolide	Sesquiterpene lactone; known to induce ROS and apoptosis, also linked to ferroptosis induction	CRC (induces cell death through multiple mechanisms)	[151]
Pathway Inhibitors (Experimental Tools)				
	Ferrostatin-1 (Fer-1)	Radical-trapping antioxidant; Specific inhibitor of ferroptosis	Multiple GI cancers (gold-standard experimental tool to inhibit ferroptosis)	[18]
	Liproxstatin-1 (Lip-1)	Spiroquinoxalinamine-based radical-trapping antioxidant; Potent inhibitor of ferroptosis	Multiple GI cancers (potent experimental tool to inhibit ferroptosis)	[29]
	Deferoxamine (DFO)	Iron chelator; Reduces the labile iron pool, thereby inhibiting ferroptosis	Multiple GI cancers (classic experimental tool to inhibit ferroptosis)	[18]
	YKL-05–099	Specific inhibitor of Salt-Inducible Kinase 1 (SIK1)	PDAC (reverses ferroptosis resistance by targeting the SIK1-HDAC5-STAT6 axis)	[120]
	Tetrathiomolybdate (TM)	Copper chelator; Reduces systemic and intracellular copper levels, inhibiting cuproplasia	Multiple cancer models (used to study the effects of copper depletion)	[54]

The induction of cuproptosis primarily relies on the excessive accumulation of intracellular copper ions, particularly their toxic effects within the mitochondria [103]. Elesclomol is a potent copper ionophore that forms a complex with copper ions, facilitating their transport across membranes and specific accumulation in the mitochondria [153]. Excess copper ions in the mitochondria directly bind to lipoylated proteins of the tricarboxylic acid (TCA) cycle, such as the E2 subunit of the pyruvate dehydrogenase complex (DLAT), causing these proteins to aggregate and lose function while also disrupting the stability of iron-sulfur cluster proteins. This triggers proteotoxic stress, ultimately leading to cell death [49].

Among small molecules, the combination of disulfiram (DSF) and copper (DSF-Cu) exemplifies a dual-induction strategy, demonstrating a powerful potential to trigger both ferroptosis and cuproptosis [154]. DSF itself is a copper ionophore that efficiently delivers copper ions into the cell, thereby initiating the cuproptosis program [153]. More critically, DSF and its metabolites can effectively deplete the cell's GSH reserves [138]. GSH depletion acts as a central hub connecting the two death modalities in this process. On one hand, the decline in GSH levels weakens the cell's ability to chelate and detoxify copper ions, greatly enhancing the effect of cuproptosis [153]. On the other hand, GSH depletion directly leads to the inactivation of GPX4, rendering the cell unable to eliminate lipid peroxides and thus initiating the ferroptosis program [61]. This mechanistic synergy, which involves simultaneously striking two key cellular pathways with a single drug combination, not only enhances anti-tumor efficacy but also offers a new approach to overcoming the adaptive resistance that may arise from targeting a single pathway [115].

Although small-molecule compounds have shown great potential in mechanistic studies and preliminary applications, their limitations, including solubility, targeting specificity, and bioavailability, have become increasingly apparent. To overcome these obstacles, nanotechnology has provided revolutionary solutions for achieving efficient and precise drug delivery.

### Nanoplatforms overcome delivery barriers for therapeutic induction

Nanotechnology provides platforms for the delivery of ferroptosis and cuproptosis inducers. Through specific engineering, nanodelivery systems can address the limitations of small-molecule drugs, including issues with solubility, bioavailability, and targeting [155]. The clinical translation of these nanoplatforms, however, is contingent on a critical evaluation of their design and performance, as existing systems face several challenges.

In terms of targeting specificity, although the enhanced permeability and retention (EPR) effect provides a theoretical basis for passive targeting of nanomedicines, its significant heterogeneity across different tumor types, and even within the same tumor, significantly limits its universality and efficiency [156]. The dense extracellular matrix (ECM) and high interstitial fluid pressure, characteristic features of solid tumors (especially pancreatic cancer), constitute physical barriers that hinder the effective penetration and uniform distribution of nanoparticles [157]. To overcome this limitation, active targeting strategies have emerged, involving the modification of nanocarrier surfaces with specific ligands to enhance binding to tumor cells. However, the heterogeneous expression of target receptors poses a challenge that active targeting strategies must address. Regarding drug-loading capacity, different platforms vary significantly. Platforms with high porosity, such as metal–organic frameworks (MOFs), exhibit excellent drug-loading capabilities [158], whereas liposomes and polymer micelles may face challenges with loading efficiency [155]. The controllability of the release mechanism is key to determining the efficacy of nanomedicines. An ideal release mechanism should respond to specific signals in the tumor microenvironment (TME), such as low pH or high GSH concentration, to achieve "smart" drug release [159], but precise regulation of release kinetics remains challenging. Biocompatibility and potential toxicity are core considerations for the clinical translation of all nanomaterials. While many organic materials (such as lipids and polymers) have good biocompatibility, the long-term safety of inorganic materials (especially nanozymes or MOFs containing heavy metals) requires careful evaluation, as metal ion leakage can cause systemic toxicity [160]. Finally, the influence of the TME itself on the efficacy of nanotherapeutics cannot be ignored. For example, high levels of GSH in the TME not only serve as a key defense line for tumor cells against oxidative stress and ferroptosis but can also directly chelate and inactivate specific metal ions (such as Cu^2^⁺), thereby weakening the effectiveness of therapeutic strategies based on metal-catalyzed reactions [161]. Therefore, a successful nanomedicine design must consider the complexity of the TME as both a barrier and a trigger to be exploited.

To address the aforementioned challenges, the field has rapidly evolved from simple drug carriers to intelligent, multifunctional theranostic platforms. These next-generation systems not only aim to deliver payloads but also to actively sense and respond to the unique biochemical landscape of the TME, act as catalysts to amplify therapeutic effects in situ, and even integrate with biological systems to create complex bio-hybrid therapies. This section will systematically elaborate on the design strategies and latest advancements in nanodelivery systems for inducing ferroptosis, cuproptosis, and their synergistic effects.

#### Single-pathway inducing nanoplatforms

Before exploring synergistic therapies, it is essential to understand the fundamental principles of nanoplatforms designed to precisely induce a single death pathway. These platforms lay the groundwork for the development of more complex systems.

The core strategy of iron-based nanoplatforms is to increase the intracellular labile iron pool (LIP) in tumors, which acts as a catalyst for the Fenton reaction. This reaction converts intracellular hydrogen peroxide (H_2_​O_2_​) into highly toxic hydroxyl radicals (•OH), ultimately triggering a chain reaction of lipid peroxidation that drives ferroptosis [157].

Magnetic iron oxide nanoparticles (MIONs), most commonly in the form of superparamagnetic iron oxide nanoparticles (SPIOs, Fe_3_​O_4_​ NPs), are a cornerstone in this field. These materials possess excellent dual functionality: on one hand, they are ideal T2-weighted magnetic resonance imaging (MRI) contrast agents, enabling non-invasive monitoring of nanodrug distribution and tumor accumulation; on the other hand, they can degrade in the acidic tumor microenvironment to release Fe^2^^+^/Fe^3^^+^, directly providing catalysts for the Fenton reaction [162]. This theranostic property provides a powerful tool for achieving precise and monitorable ferroptosis therapy. For example, one study designed a platelet membrane (PM)-camouflaged nanoplatform (PM@ESL NPs) co-loaded with SPIO, lactate oxidase (LOX), and the ferroptosis inducer erastin. This platform could not only be guided by MRI but its loaded LOX could also consume lactate in hepatocellular carcinoma tissue to produce H_2_​O_2_​, which was then catalyzed by SPIO to generate •OH, thereby greatly enhancing sensitivity to ferroptosis [162]. Another study co-encapsulated arsenic trioxide (ATO) and Fe3​O4​ within an HCC cell membrane (AFN@CM), utilizing the homologous targeting of the cancer cell membrane for precise delivery and efficiently inducing ferroptosis through the synergistic action of ATO and iron ions [163].

Metal–organic frameworks (MOFs), porous crystalline materials self-assembled from metal ions and organic ligands, have garnered significant attention due to their high drug-loading capacity and TME-responsive degradation. Iron-based MOFs can disintegrate in the acidic tumor microenvironment, simultaneously releasing iron ions and pre-loaded chemotherapy drugs. For instance, a sorafenib-loaded Fe-MOF nanoplatform (Sor@Fe-MOF) in an HCC model not only enhanced ferroptosis by releasing iron ions and sorafenib to downregulate GPX4 and SLC7A11 expression synergistically but also effectively remodeled the tumor immune microenvironment to activate anti-tumor immunity [158].

Furthermore, nanozymes with enzyme-like activities have opened new avenues for ferroptosis therapy. For example, pyrite nanozymes have been shown to possess excellent dual peroxidase (POD) and glutathione oxidase (GSHOx) activities. They can not only catalyze endogenous H_2_​O_2_​ to generate •OH but also directly consume GSH, thereby dismantling the GPX4 defense system and synergistically inducing apoptosis and ferroptosis [164]. These designs demonstrate that iron-based nanoplatforms have evolved beyond the role of mere "iron supplements" to become "signal amplifiers" that can potentiate the oxidative stress generated by other therapies (such as radiotherapy and photodynamic therapy), forming the core hub of multimodal synergistic treatments.

Unlike ferroptosis, which relies on the Fenton reaction, the core mechanism of cuproptosis is the direct binding of excess copper ions (particularly Cu^+^) to lipoylated proteins in the tricarboxylic acid (TCA) cycle within mitochondria, such as DLAT. This leads to their abnormal oligomerization and aggregation, induces the loss of iron-sulfur cluster proteins, and ultimately triggers lethal proteotoxic stress [160]. Therefore, the design of copper-based nanoplatforms focuses on efficiently delivering copper ions to tumor cells, particularly to the mitochondria.

Copper oxide (CuO/Cu₂O) nanoparticles are among the most extensively studied copper ion donors. They are relatively stable at physiological pH but dissolve in the acidic and high-GSH microenvironment of tumors, releasing bioactive copper ions [165]. For example, an engineered microbial-nanohybrid (E. coli@Cu₂O) can target colon tumors and, in the presence of abundant hydrogen sulfide within the tumor, transform in situ into copper sulfide, which exhibits excellent near-infrared II photothermal conversion efficiency. This achieves photothermal therapy while the released copper ions induce cuproptosis and ferroptosis [166].

Copper-based MOFs (Cu-MOFs) and nanoscale coordination polymers (NCPs) offer more refined control over copper ion release. For instance, a "three-in-one" Cu-MOF nanozyme (Cu-PrIm) in a colorectal cancer model not only induced cuproptosis and apoptosis by releasing copper ions but also degraded hypoxia-inducible factor-1α (HIF-1α), thereby reversing chemoresistance [167]. A more sophisticated design involves constructing mitochondria-targeting NCPs (Cu/TI), which are self-assembled from copper ions and a mitochondria-targeting ligand. This ensures that copper ions are precisely delivered to the site of cuproptosis execution, greatly enhancing therapeutic selectivity and efficiency, and can synergistically downregulate PD-L1 expression, providing an opportunity for combined immunotherapy [168].

An advanced strategy in copper-based nanotherapy involves actively interfering with the cell's copper homeostasis mechanisms. Normal cells have efficient copper efflux pumps (such as ATP7A/B) to prevent copper toxicity. One study utilized bacterial outer membrane vesicles (OMVs) to co-deliver siRNA targeting the copper chaperone protein Atox1 and the copper ionophore elesclomol (siAtox1/ES@OMV). By silencing the gene to inhibit copper efflux while using the ionophore to promote copper influx, this strategy traps copper ions within tumor cells, greatly amplifying the effect of cuproptosis [169]. These designs cleverly exploit the dual threat of copper ions: first, the proteotoxicity produced by direct binding to lipoylated proteins to induce cuproptosis, and second, their participation in Fenton-like reactions to generate ROS and consume GSH, which not only exacerbates oxidative stress but also sets the stage for synergistic induction of ferroptosis.

#### Synergistic nanodelivery systems

Building on the understanding of single-pathway nanoplatforms, researchers have developed more complex bimetallic or hybrid nanosystems. These systems aim to achieve a "dual-strike" strategy by simultaneously or sequentially activating both ferroptosis and cuproptosis pathways, seeking a synergistic effect greater than the sum of its parts (1 + 1 > 2) and effectively overcoming the adaptive resistance that tumor cells may develop.

The core mechanism of these synergistic strategies often revolves around two key cellular biological nodes: GSH metabolism and mitochondrial function. GSH is both a necessary cofactor for GPX4 to inhibit ferroptosis and the primary buffer for chelating copper ions in the cell. Therefore, any strategy that can effectively deplete GSH will simultaneously weaken the cell's defense against both death modalities [161]. For example, a modified copper oxide nanozyme (MitCuOHA) was designed to have dual cysteine oxidase and glutathione oxidase activity. It targets mitochondria and catalytically depletes cysteine and GSH, which have two direct consequences: GPX4 inactivation triggers ferroptosis, and copper ions dissociate to attack lipoylated proteins, triggering cuproptosis. Thus, a single nanozyme achieves the simultaneous activation of both death pathways [170].

Mitochondria, as the execution site for cuproptosis and a signal amplifier for ferroptosis, serve as another key platform connecting the two pathways. At the mitochondrial level, the two pathways form a reinforcing cycle of mutual amplification. The core event of cuproptosis, the aggregation of lipoylated proteins and the degradation of iron-sulfur cluster proteins, directly releases iron ions, expanding the LIP and providing "fuel" for ferroptosis. At the same time, the destruction of iron-sulfur clusters leads to the collapse of the electron transport chain, causing a massive leakage of ROS that initiates lipid peroxidation [171]. Based on this principle, researchers have designed various bimetallic nanosystems. For example, a core–shell Cu₂O@Mn₃Cu₃O₈ nanozyme releases copper and manganese ions in the TME, and its GSHOx-like activity depletes GSH, synergistically promoting both cuproptosis and ferroptosis [172]. Another example is a heterojunction platform integrating an iron-based MOF (MIL-88B) and mitochondria-targeting Cu₁.₈S nanodots (MIL-Cu₁.₈S-TPP/FA), which can precisely deliver copper ions to mitochondria to induce cuproptosis. This process, in turn, promotes the occurrence of ferroptosis, forming an efficient killing cascade [173].

Besides leveraging intrinsic synergistic mechanisms, co-delivering inducers of different pathways is also a direct and effective strategy. For example, co-encapsulating copper ions and the classic ferroptosis inducer erastin in a nanocarrier (CuP/Er). Erastin depletes GSH by inhibiting cystine uptake, which not only directly initiates ferroptosis but also greatly enhances the cell's sensitivity to copper toxicity, thereby significantly amplifying the effect of cuproptosis [138]. This strategy, which synergistically attacks multiple core life centers such as cellular redox homeostasis, proteostasis, and organelle integrity, aims to push tumor cells to an irreversible ‘tipping point of death,’ thereby effectively overcoming the adaptive resistance that may arise from single-agent therapies. Representative synergistic nanoplatform designs and their applications in GI cancers are summarized in Table 3.

**Table 3:** Nanoplatforms for inducing ferroptosis and cuproptosis

Nanoplatform	Core Composition & Mechanism	Key Features/Functionality	Target Cancer Model	Ref
Ferroptosis-Inducing Platforms				
PPR@LVN/IR780	PLGA-PEG nanospheres co-loaded with lenvatinib (LVN) and photosensitizer IR780, modified with cRGD peptide	Active targeting via cRGD. Upon laser irradiation, PDT/PTT from IR780 synergizes with LVN to induce both ferroptosis and apoptosis	HCC	[174]
FCE NPs	Acid-responsive nanoplatform of ferric ions and Chlorin e6 (Ce6) loaded with evodiamine	TME-activated PDT generates ROS to deplete GSH. Released Fe(III) catalyzes Fenton reaction. Evodiamine inhibits GPX4 to amplify ferroptosis	CRC	[175]
FVIO + SRF	Combination of ferrimagnetic vortex-domain iron oxide nanorings (FVIO) and sorafenib (SRF)	FVIO acts as an iron source and a magnetic hyperthermia agent. Under an alternating magnetic field, localized heating enhances the Fenton reaction, amplifying SRF-induced ferroptosis	HCC	[176]
O(3)/PFTBA@LIP@Gel	Ozone-enriched perfluorotributylamine (PFTBA) nanoemulsion encapsulated in a thermoresponsive hydrogel	Sprayable gel for postsurgical application. It releases ozone, which induces both ferroptosis and apoptosis by regulating GPX4/ACSL4 and causing lipid peroxidation	HCC	[177]
DECaNPs	Calcium carbonate (CaCO(3))-based nanoparticles co-encapsulating doxorubicin (DOX) and erianin	Induces calcium overload, neutralizes acidic TME, and generates oxidative stress, leading to a hybrid ferroptosis/apoptosis pathway and immunogenic cell death	HCC	[178]
FCRM	Bionic 2D FeS nanoplatform with high CRISPR/Cas9 loading capacity	pH-responsive release of CRISPR/Cas9 (to downregulate Survivin) and Fe(2 +) (to induce ferroptosis), synergistically overcoming photothermal resistance	GC	[179]
Photosensitive LNPs	Lipid nanoparticles (LNPs) co-delivering FSP1 siRNA and the photosensitizer Verteporfin	Light-activated PDT generates oxidative stress, which synergizes with FSP1 gene silencing to induce potent ferroptosis and immunogenic cell death for enhanced immunotherapy	CRC	[180]
HC-GO	High Carbonyl Graphene Oxide	Suppresses the System Xc-/GSH/GPX4 pathway, leading to mitochondrial damage, iron accumulation, and ROS production, thereby inducing ferroptosis	CRC	[181]
Cuproptosis-Inducing Platforms				
Elesclomol-Cu Hydrogel	Sodium alginate hydrogel consisting of elesclomol-Cu and galactose	Implantable hydrogel for controlled release of elesclomol-Cu, which induces DLAT oligomerization and cuproptosis. It also abrogates radiation-induced PD-L1 upregulation	CRC	[182]
Cu(5.4)O NPs	Multifunctional Cu-based nanoenzyme (Cu(5.4)O)	Acts as a "three-level nanoparticle rocket" combining photothermal therapy, inflammation modulation (ROS scavenging), and cuproptosis induction via Cu(+) release	CRC	[183]
Lyc@GA-Cu Gel	Self-assembled hydrogel from natural small molecules: glycyrrhizic acid (GA), lycorine (Lyc), and Cu(2^+^)	Acts as a copper ionophore to induce cuproptosis, which in turn generates massive ROS, triggering a broader PANoptosis (apoptosis, pyroptosis, necroptosis)	CRC	[184]
TSF@ES-Cu NPs	Tussah silk fibroin nanoparticles loaded with Elesclomol (ES) and Cu(2^+^)	Utilizes a natural polymer carrier for ES-Cu delivery to induce cuproptosis, which reshapes the tumor microenvironment and enhances immunotherapy	PDAC	[185]
Cu(I) NP	Stimulus-responsive copper complex nanoparticles	Releases copper complexes in response to stimuli, causing mitochondrial dysfunction and DLAT aggregation to induce cuproptosis and elicit robust immune responses	PDAC	[186]
Synergistic Ferroptosis-Cuproptosis Platforms				
HA-ZCu	Cu(0)-based nanoparticles	Induces cuproptosis, which disrupts mitochondrial function and depletes intracellular GSH, thereby enhancing a secondary ferroptosis response	HCC	[187]
DFMTCH NPs	MIL-100 MOF loaded with doxorubicin (DOX) and disulfiram (DSF), coated with a Cu-tannic acid network and HA	Multifunctional platform for combined chemotherapy, PTT, and CDT. It generates ROS and consumes GSH, inducing a trimodal cuproptosis/ferroptosis/apoptosis cell death	CRC	[154]

#### Intelligent and biomimetic nano-strategies

Advanced nanotherapeutic strategies include "smart" systems that respond to specific signals within the TME and "biomimetic" systems that incorporate biological components. The design of smart nanocarriers is based on utilizing the physicochemical differences between the TME and normal tissues, such as pH, redox potential, and enzyme profiles, to trigger drug activation and release specifically at the tumor site [188]. For instance, the weakly acidic environment of tumor tissue (pH ≈ 6.5–6.8) can be used to trigger the decomposition of pH-sensitive materials, such as calcium carbonate (CaCO_3_​) nanoparticles. One ingenious design involves using CaCO_3_​ to encapsulate copper peroxide (CuO_2_​) and coating it with a lipid layer (CaCO_3_​@CuO_2_​@L). In the acidic tumor environment, the CaCO3​ core dissolves, releasing CuO_2_​ to induce cuproptosis while simultaneously consuming protons and producing oxygen, thereby favorably modulating the TME [189]. Similarly, the high concentration of GSH within tumor cells can be used as a "trigger" for drug release by reducing and cleaving disulfide bonds (-S–S-) in the nanocarrier [161]. Furthermore, targeting the high expression of H2​S in colorectal cancer, researchers have designed an H2​S-responsive nanoplatform (TCuH) that reacts with H2​S to form the photothermal agent Cu9​S8​ in situ and release chemotherapy drugs, achieving multiple synergistic therapeutic effects [190]. These innovative systems embody a "logic-gated" design philosophy, where the therapeutic function is activated only when multiple TME conditions are met, greatly enhancing treatment precision.

Concurrently, biomimetic strategies aim to address a core challenge in nanomedicine, the bio-nano interface, by "camouflaging" synthetic nanoparticles with natural biological membranes to avoid recognition and clearance by the immune system. For example, coating nanoparticles with cancer cell membranes (such as AFN@CM [163] and Len/FePt@CMP NPs [191]) utilizes the principle of "homologous targeting," where adhesion molecules on the membrane surface guide the nanoparticles to recognize and bind to tumor cells of the exact origin, while also evading phagocytosis by the reticuloendothelial system (RES). Using platelet membranes (PM) to coat nanoparticles (such as PM@ESL NPs [162]) leverages the natural tendency of platelets to accumulate at sites of inflammation, like tumors, achieving efficient targeted delivery. Additionally, using cell-secreted exosomes or bacteria-derived outer membrane vesicles (OMVs) as natural nanocarriers offers excellent biocompatibility and potential immunomodulatory functions, providing new avenues for constructing multifunctional therapeutic platforms [169]. These biomimetic strategies, by skillfully integrating the precise engineering of materials science with the complex functions of cell biology, greatly enhance the in vivo performance of nanomedicines and are a crucial step toward their clinical application. Future directions may involve combining "smart-responsive" cores with "biomimetic" shells to construct next-generation intelligent hybrid nanomedical systems capable of multi-level, programmed responses at the systemic, tissue, cellular, and even subcellular levels.

### Inducing cell death synergizes with conventional cancer therapies

Inducing ferroptosis and cuproptosis can not only directly kill tumor cells but also produce powerful synergistic effects with traditional anticancer treatments, such as immunotherapy, radiotherapy, and chemotherapy, by modulating the tumor microenvironment and cellular signaling pathways [192]. This synergy is primarily mediated by the induction of immunogenic cell death (ICD), which can convert immunologically non-responsive tumors into tumors with active immune cell infiltration [193].

In synergy with immunotherapy, dying tumor cells undergoing ferroptosis or cuproptosis release a series of damage-associated molecular patterns (DAMPs), including ATP, calreticulin (CRT), and high-mobility group box 1 (HMGB1) [194, 195]. These DAMPs act as "danger signals" that are recognized by antigen-presenting cells (APCs), particularly dendritic cells (DCs), thereby promoting DC maturation and their antigen-presenting capacity [196]. Mature DCs then migrate to lymph nodes, where they effectively present tumor antigens to naive T cells, activating and expanding tumor-specific cytotoxic T lymphocytes (CTLs) [197]. These activated CTLs subsequently infiltrate the tumor tissue and specifically kill tumor cells. Therefore, initiating ICD by inducing ferroptosis or cuproptosis can effectively enhance the efficacy of immune checkpoint blockade (ICB), such as anti-PD-1/PD-L1 antibodies [198]. For example, in a colorectal cancer model, ferroptosis induced by the natural product Icariin (ICA) activated CD8 + T cells to secrete IFN-γ, thereby synergistically enhancing the effect of anti-PD-1 therapy [148]. Similarly, in hepatocellular carcinoma, a nanoplatform that induces cuproptosis also promoted the infiltration of CTLs, showing significant anti-tumor synergy when combined with anti-PD-1 therapy [196].

In synergy with radiotherapy (RT), the amplification of oxidative stress is the key mechanism [199]. Radiotherapy itself generates a large amount of ROS in cells through ionizing radiation, which damages DNA and induces cell death. However, tumor cells often possess robust antioxidant systems that enable them to withstand this damage [108]. Ferroptosis or cuproptosis inducers severely weaken the antioxidant defenses of tumor cells by inhibiting GPX4 or depleting GSH, allowing the ROS generated by radiotherapy to cause more lasting and extensive damage, thereby significantly enhancing the killing effect of RT [112]. Studies have shown that radiotherapy itself can induce ferroptosis by downregulating the expression of SLC7A11. This effect can be amplified by IFN-γ, providing a new molecular link for the synergy between radiotherapy and immunotherapy [200]. Furthermore, radiotherapy can directly induce cuproptosis by upregulating the expression of copper transporters (like CTR1) and increasing mitochondrial copper ion concentration. This provides a direct molecular basis for the combined use of radiotherapy and cuproptosis inducers (like Elesclomol) and has been confirmed to have a sensitizing effect in an esophageal squamous cell carcinoma model [201].

Synergy with chemotherapy mainly focuses on overcoming drug resistance. The resistance mechanisms to many chemotherapy drugs are closely related to the enhancement of the antioxidant capacity of tumor cells [103]. For instance, in colorectal cancer, cells that have developed resistance to platinum-based drugs, such as cisplatin or oxaliplatin, often exhibit higher levels of GSH and more vigorous GPX4 activity [35, 202]. By using ferroptosis inducers such as SSZ or RSL3, this resistant phenotype can be effectively reversed, thereby restoring the sensitivity of tumor cells to chemotherapy drugs [35]. In gastric cancer, a traditional Chinese medicine formula can reverse cisplatin resistance by inducing ferroptosis via the AKT/GSK3β/NRF2/GPX4 axis [93]. In pancreatic cancer, knocking out the ACOT8 gene promotes ferroptosis and increases the tumor's chemosensitivity to gemcitabine [118]. This strategy, by targeting the metabolic reprogramming that tumor cells undergo to adapt to chemotherapy stress, offers a new avenue for treating recurrent and refractory tumors.

### Emerging clinical strategies target ferroptosis and cuproptosis

The clinical translation of therapies targeting ferroptosis and cuproptosis in gastrointestinal (GI) malignancies is in early stages, despite extensive preclinical evidence. Current clinical trials are proceeding through two main strategies: repurposing existing drugs with established safety profiles and developing novel agents specifically designed to modulate these cell death pathways. This approach combines the use of established pharmaceuticals with investment in new, mechanism-driven therapeutics.

The drug repurposing strategy has provided the earliest clinical insights. Sorafenib, a multikinase inhibitor approved for hepatocellular carcinoma (HCC), was retrospectively found to induce ferroptosis by inhibiting System Xc⁻. This dual mechanism has prompted further investigation, such as in trial NCT02989870, which explored its synergy with stereotactic body radiation therapy (SBRT) in HCC, and NCT05068752, which is evaluating its combination with vemurafenib in KRAS-mutant pancreatic cancer. Similarly, sulfasalazine, a long-standing anti-inflammatory drug and a canonical System Xc⁻ inhibitor, is being formally tested in combination with standard chemotherapy for metastatic colorectal cancer (NCT06134388). Another notable example is the antimalarial drug artesunate, which induces ferroptosis by generating reactive oxygen species (ROS). The ongoing NeoART trial (NCT02633098) is strategically significant as it assesses artesunate in the neoadjuvant setting for colorectal cancer, aiming to improve surgical outcomes and reduce recurrence by targeting the disease at an earlier stage. In the realm of cuproptosis, the combination of the alcohol-aversion drug disulfiram with copper has been the cornerstone of clinical investigation. The Phase 1 trial NCT00742911, the foundational trial, established the safety and tolerability of this combination in patients with liver metastases but observed no objective responses, highlighting the central challenge of translating this strategy into potent clinical efficacy. Subsequent studies, such as NCT03714555 in metastatic pancreatic cancer, have continued to explore this combination in specific GI tumor contexts.

To overcome the potential limitations in potency and specificity of repurposed drugs, the field is increasingly focusing on novel and targeted therapeutic strategies. These approaches aim to engage the core machinery of ferroptosis and cuproptosis more directly and powerfully. One prominent example is eprenetapopt (APR-246), a novel small molecule that not only reactivates mutant p53 but also potently depletes cellular glutathione (GSH), thereby inducing ferroptosis robustly. Its investigation in the NCT04383938 trial, which combines eprenetapopt with the immune checkpoint inhibitor pembrolizumab and includes an expansion cohort for advanced gastric cancer, exemplifies a modern strategy to test the synergy between ferroptosis-induced immunogenic cell death and immunotherapy. A more direct approach to modulating the GSH axis is demonstrated in trial NCT04205357, which combines buthionine sulfoximine, a specific inhibitor of GSH synthesis, with gemcitabine in patients with pancreatic ductal adenocarcinoma. Perhaps the most forward-looking developments are in the realm of purpose-built nanomedicine. These platforms are engineered to address the fundamental challenge of delivering therapeutic metal ions, such as iron, specifically to tumors while minimizing systemic toxicity. A landmark study in this area is the recently completed Phase 1 trial NCT06048367, which evaluated the safety and preliminary efficacy of intratumoral injection of Carbon Nanoparticle-Loaded Iron in patients with advanced solid tumors, including various GI cancers. This trial represents a critical first-in-human validation of a nanomedicine specifically designed to induce ferroptosis, marking a pivotal step from preclinical concept to clinical reality. The current landscape of representative clinical trials investigating these strategies in gastrointestinal cancers is summarized in Table 4.

**Table 4:** Representative clinical trials of therapies targeting ferroptosis or cuproptosis in GI cancers

rial ID	Drug/Intervention	Mechanism	Cancer Type	Phase	Status
Cuproptosis-Targeting Strategies					
NCT00742911	Disulfiram/Copper Gluconate	Cuproptosis (Copper Ionophore)	Advanced Solid Tumors (Liver Mets)	Phase 1	Completed
NCT03714555	Disulfiram/Copper Gluconate	Cuproptosis (Copper Ionophore)	Metastatic Pancreatic Cancer	Phase 2	Completed
Ferroptosis-Targeting Strategies: Drug Repurposing					
NCT02989870	Sorafenib/SBRT/Bavituximab	Ferroptosis (System Xc⁻ Inhibition)/Radiosensitization	Unresectable Hepatocellular Carcinoma	Phase 1	Terminated
NCT05068752	Sorafenib/Vemurafenib	Ferroptosis (System Xc⁻ Inhibition)	KRAS-mutant Pancreatic Cancer	Phase 2	Recruiting
NCT06134388	Sulfasalazine/FOLFOX	Ferroptosis (System Xc⁻ Inhibition)	Metastatic Colorectal Cancer	Phase 2	Recruiting
NCT02633098	Artesunate	Ferroptosis (ROS Induction)	Colorectal Cancer (Neoadjuvant)	Phase 2	Recruiting
Ferroptosis-Targeting Strategies: Novel and Targeted Agents					
NCT04383938	Eprenetapopt (APR-246)/Pembrolizumab	Ferroptosis (GSH Depletion)/p53 Activation	Advanced Solid Tumors (incl. Gastric Cancer)	Phase 1/2	Recruiting
NCT04205357	Buthionine Sulfoximine/Gemcitabine	Ferroptosis (GSH Synthesis Inhibition)	Pancreatic Ductal Adenocarcinoma	Phase 1	Recruiting
NCT06048367	Carbon Nanoparticle-Loaded Iron	Ferroptosis (Intratumoral Iron Delivery)	Advanced Solid Tumors (incl. GI Cancers)	Phase 1	Completed

Data Source: Information was compiled from the U.S. National Library of Medicine's clinical trial registry (ClinicalTrials.gov). Each trial was accessed and verified using its unique NCT identifier. The status of each trial is reported as of the latest verification date available in the registry

*Abbreviations*: *APR-246* Eprenetapopt, *FOLFOX* Combination chemotherapy regimen of Folinic acid (leucovorin), Fluorouracil, and Oxaliplatin, *GI* Gastrointestinal, *GSH* Glutathione, *Mets* Metastases, *NCT* National Clinical Trial, *ROS* Reactive Oxygen Species, *SBRT* Stereotactic Body Radiation Therapy

## Biomarkers and translational pathways

The clinical translation of therapies targeting ferroptosis and cuproptosis requires robust biomarkers for predicting therapeutic response, monitoring efficacy, and stratifying patients for personalized treatment. Current research primarily utilizes multi-omics data, especially transcriptomics, to identify gene expression signatures associated with these cell death pathways. This has led to the construction of sophisticated molecular subtyping and risk-scoring models that can predict clinical prognosis and therapeutic sensitivity. These bioinformatics-driven studies have provided unprecedented new insights into the roles of ferroptosis and cuproptosis in tumor heterogeneity and their complex interactions with the tumor microenvironment (TME), thereby laying a solid theoretical foundation for future clinical applications.

### Molecular subtypes via metal-dependent death signatures

Patient stratification based on the expression profiles of cuproptosis- and ferroptosis-related genes is being used to investigate the heterogeneity of GI tumors. Unsupervised consensus clustering of cuproptosis-related genes (CRGs) has been employed to identify previously unrecognized tumor subtypes. These subtypes are not randomly distributed but show consistent and significant associations with distinct clinical outcomes and TME characteristics [203–205].

In gastric cancer (GC), clustering analysis based on CRG expression profiles has successfully identified multiple molecular subtypes. Crucially, these subtypes are closely related to prognosis; for instance, studies have found that subtypes characterized by high expression of CRGs paradoxically have a better prognosis [206, 207]. This finding challenges the simple assumption that an active cell death pathway is always beneficial for tumor suppression, suggesting that the context and specific mechanisms of cuproptosis activation are critical. Similarly, in colorectal cancer (CRC), two distinct cuproptosis-related molecular subtypes have been identified, which show significant differences in clinicopathological features, overall survival, and TME activity [205, 208]. One study directly linked three cuproptosis patterns to three distinct immune infiltration phenotypes, namely immune-desert, immune-inflamed, and immune-excluded, thereby tightly connecting cuproptosis regulation with the tumor's immune microenvironment [208].

Even in pancreatic ductal adenocarcinoma (PDAC), known for its extreme heterogeneity and therapeutic resistance, CRG-based clustering analysis has identified three unique cuproptosis subtypes. These subtypes exhibit significant differences in patient prognosis and TME cell infiltration, highlighting the immense potential of cuproptosis-based stratification strategies even in the most challenging gastrointestinal malignancies [204]. Research in cholangiocarcinoma (CCA) has also found two CRG-related clusters with opposing survival times, further validating the general applicability of this method across multiple gastrointestinal tumors [209].

A recurring and compelling theme in these findings is the "prognostic paradox" of cuproptosis activity. Intuitively, high expression of CRGs might imply a greater potential for cuproptosis, which should lead to more tumor cell death and a better prognosis. However, multiple studies consistently show that high expression levels of CRGs are associated with better patient survival [206, 207]. There may be a more profound biological logic behind this phenomenon. High CRG expression may not represent a continuously active cell death process but rather reflect a specific metabolic state that is highly dependent on mitochondrial respiration. This metabolic phenotype itself may be less aggressive or have a weaker immune escape capability, leading to a better clinical outcome independent of the actual cuproptosis induction process. Another possibility is that this "primed-for-death" state makes the tumor extremely sensitive to endogenous copper fluctuations or therapeutic interventions, ultimately leading to its effective control.

Furthermore, molecular subtyping has become a bridge connecting cell death and tumor immunology. The consistent association between cuproptosis-defined subtypes and the TME landscape [205, 208] implies that the regulation of copper metabolism is not an isolated cellular process; it is fundamentally linked to the mechanisms that shape tumor-immune interactions. The molecular machinery that regulates cuproptosis likely determines the tumor's potential for immunogenic cell death (ICD). A subtype with high expression of key cuproptosis execution proteins (such as FDX1, DLAT) may be more prone to ICD when stressed, thereby shaping an "immune-inflamed" microenvironment over time. Conversely, a subtype that has evolved to suppress the expression of these genes would be resistant to ICD, ultimately forming an "immune-desert" phenotype. Therefore, cuproptosis subtyping is not only a prognostic tool but also a functional interpretation of the tumor's immunogenic potential.

### Prognostic signatures and risk models

Multi-gene prognostic signature models are being constructed as an extension of molecular subtyping. These models utilize machine learning algorithms to generate a quantitative risk score from gene expression data, enabling the prediction of patient outcomes. A common framework involves an initial screen for prognosis-related genes using univariate Cox regression, followed by variable selection with the least absolute shrinkage and selection operator (LASSO) regression, and final model construction through multivariate Cox analysis [210–212]. This rigorous statistical approach aims to prevent model overfitting and enhance its generalizability.

Signature models based on protein-coding genes have been validated in various gastrointestinal tumors. In cholangiocarcinoma (CCA), a 4-gene signature (ATP7A, FDX1, DBT, LIAS) was established as an independent prognostic factor [212], while another study constructed a signature model containing 10 genes [213]. In colorectal cancer (CRC), multiple models have also been developed, including a 5-gene signature [214] and a 12-gene signature [215]. In gastric cancer (GC), a 7-gene prognostic model was established using the random forest method [216].

A significant and recent development is the shift in research focus towards constructing models based on CRLs. Due to their high stability and tissue specificity, lncRNAs may offer superior potential as biomarkers. In colon adenocarcinoma (COAD), multiple studies have successfully constructed prognostic signatures based on CRLs, identifying models containing 5, 13, and 15 lncRNAs, respectively [211, 217, 218]. These models consistently demonstrate high predictive accuracy (area under the receiver operating characteristic curve, AUC values typically > 0.75) and can serve as independent prognostic factors[211, 219]. In pancreatic cancer (PAAD), a 5-lncRNA signature model was validated in both the TCGA and ICGC cohorts [220]. Other studies have further confirmed the value of CRLs in prognostic assessment [221]. In esophageal cancer (ESCA), a 6-CRL model was constructed to predict prognosis [222].

Some of the most innovative models integrate genes from multiple cell death pathways or other related biological processes. In esophageal squamous cell carcinoma (ESCC), a prognostic model integrating cuproptosis, ferroptosis, and immune-related genes has been constructed, thereby creating a more comprehensive signature that reflects the tumor's cell death and immune landscape [223, 224]. In colorectal cancer, one study developed a signature model based on cuproptosis-related RNA methylation regulators, directly linking RNA epigenetics with cuproptosis and prognosis [225].

Research has increasingly shifted from protein-coding genes to their regulators, such as cuproptosis-related lncRNAs (CRLs). This focus is based on the biological function of lncRNAs as upstream regulators that can control the expression of multiple protein-coding genes. Consequently, signature models based on lncRNAs may offer greater robustness compared to those based on downstream effector genes.

Although the various signature models in different cancer types contain different genes, they all functionally converge on a "tumor vulnerability index." A high-risk score always predicts a poor prognosis [211, 214, 226]. These risk models are not merely measuring the expression of random genes but are quantifying a core biological property of the tumor. A "high-risk" score may reflect that the cell has successfully suppressed endogenous cell death pathways (such as cuproptosis and ferroptosis). This suppression is a prerequisite for the tumor to achieve aggressive behaviors (such as proliferation, invasion, and therapeutic resistance). Therefore, the risk score can be seen as a proxy for the overall resilience and adaptability of the tumor, quantifying the success of cancer cells in evading death by reprogramming their metabolism, which is intrinsically linked to a more malignant phenotype and poorer prognosis. To systematically present these findings, the following table summarizes representative cuproptosis- and ferroptosis-related prognostic models in gastrointestinal tumors.

**Fig. 5:**
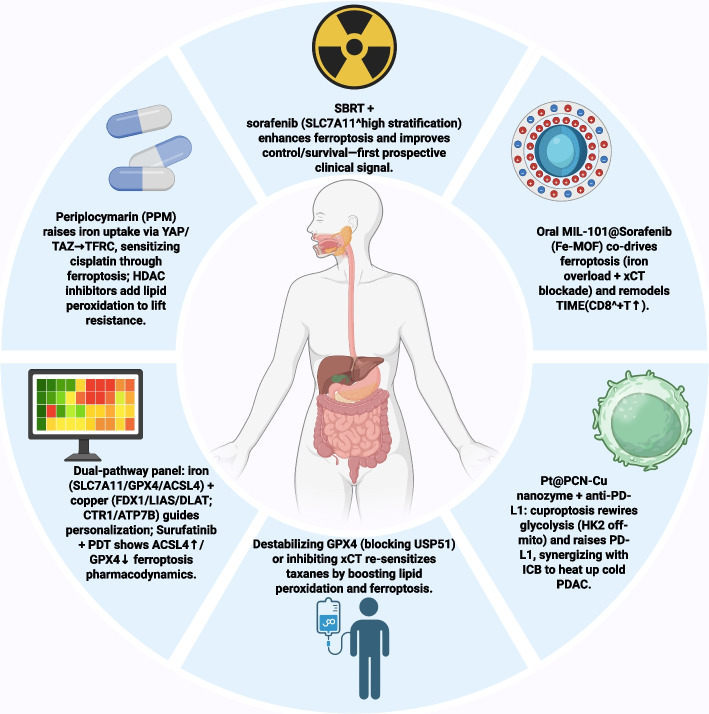
Translational blueprint for targeting ferroptosis and cuproptosis in GI cancers. This figure illustrates a multi-pronged strategy for translating the fundamental mechanisms of ferroptosis and cuproptosis into clinical applications for gastrointestinal malignancies. Key approaches include: **(i)** Synergizing with standard therapies, such as combining SBRT with sorafenib to enhance ferroptosis in high-risk patient strata [200]. **(ii)** Advanced nanodelivery systems, like oral Fe-MOF nanoparticles (MIL-101@Sorafenib) that co-deliver ferroptosis inducers and remodel the tumor immune microenvironment [158], or Pt@PCN-Cu nanozymes that induce cuproptosis to overcome immunotherapy resistance in pancreatic cancer [227]. **(iii)** Overcoming chemoresistance by using small molecules to destabilize GPX4 or inhibit System Xc⁻, thereby re-sensitizing tumors to conventional agents like taxanes [228]. **(iv)** Development of companion diagnostics, such as a dual-pathway biomarker panel assessing key proteins in both iron and copper metabolism to guide personalized treatment selection [204]. **(v)** Repurposing existing drugs, for example, using Periplocymarin (PPM) to sensitize tumors to cisplatin by modulating iron uptake and inducing ferroptosis [146]. Collectively, this blueprint integrates predictive biomarkers, innovative drug delivery, and rational combination therapies to pave the way for precision medicine targeting these metabolic vulnerabilities. Abbreviations: FDX1, ferredoxin 1; GPX4, glutathione peroxidase 4; IHC, immunohistochemistry; NCT, National Clinical Trial identifier; OS, overall survival; PFS, progression-free survival; PK/PD, pharmacokinetics/pharmacodynamics; qPCR, quantitative polymerase chain reaction; RNA-seq, RNA sequencing; ROC-AUC, receiver operating characteristic–area under the curve; RS, risk score; SLC7A11, solute carrier family 7 member 11

To visually summarize the clinical translational blueprint for targeting these emerging cell death pathways in gastrointestinal cancers, we present Fig. 5.

## Conclusion and outlook

Research on ferroptosis and cuproptosis in GI malignancies has made significant strides in recent years, clearly establishing the immense value of these non-apoptotic cell death pathways as potential anti-cancer strategies [60, 85]. Numerous in vitro and animal studies have confirmed that specifically inducing ferroptosis or cuproptosis can effectively inhibit tumor growth and overcome resistance to conventional therapies [229, 230]. Core regulators of ferroptosis, such as GPX4 and SLC7A11, are highly expressed in various GI tumors and are associated with poor prognosis; inhibiting their function can effectively trigger lipid peroxidation-mediated death in tumor cells [47, 88, 231]. Similarly, key molecules of cuproptosis, such as FDX1 and DLAT, have been shown to function as tumor suppressors in GI tumors like HCC, and activating this pathway can significantly reduce tumor cell viability [21, 105]. More importantly, profound mechanistic crosstalk exists between these two metal-dependent death processes. For example, they share GSH as a key inhibitor and mutually amplify lethal effects through mitochondrial dysfunction and Fe-S cluster destabilization, providing a solid molecular basis for developing synergistic therapeutic strategies [25, 61, 65].

Despite considerable progress, translating these basic research findings into clinical applications still faces numerous challenges and unresolved questions. First, there are currently no new drugs specifically designed to induce ferroptosis or cuproptosis that have entered clinical trials. Most current research relies on repurposing existing drugs, whose specificity, potency, and pharmacokinetic properties may not be optimal[229]. Second, the lack of biomarkers is the biggest obstacle to precision medicine. We urgently need to develop and validate biomarkers that can reliably predict which patients will respond to specific death inducers, as well as pharmacodynamic indicators that can dynamically monitor treatment efficacy [232–234]. Finally, safety issues cannot be ignored. Iron and copper are essential trace elements, and systemically disrupting their homeostasis may cause severe toxic side effects in normal tissues, especially the liver, kidneys, and nervous system. Therefore, determining a safe therapeutic window and achieving precise tumor targeting are key scientific questions that must be addressed before clinical translation [16, 59].

To overcome the aforementioned challenges and further advance the field, future basic research should focus on deeper mechanistic explorations. First, a more comprehensive understanding of the synergistic mechanisms of ferroptosis and cuproptosis is needed, particularly regarding the specific molecular nodes and optimal induction sequences in the context of the distinct genetic and metabolic backgrounds of various GI tumors. Second, efforts should be dedicated to discovering and identifying novel key molecules and signaling pathways that regulate these two death modalities, providing more high-quality targets for drug development. Third, in-depth research into how the tumor microenvironment dynamically regulates the sensitivity of tumor cells to ferroptosis and cuproptosis will provide a basis for designing more innovative therapeutic strategies. Fourth, exploring the interactions between ferroptosis, cuproptosis, and other cell death pathways (such as apoptosis, autophagy, and necroptosis) may reveal more complex cell death regulatory networks and offer new ideas for overcoming multidrug resistance.

In terms of clinical translation, future efforts should focus on several key directions. First, the development and preclinical evaluation of novel small-molecule inducers with high specificity and good druggability should be vigorously promoted. Second, developing and validating reliable companion diagnostics is a prerequisite for achieving personalized therapy, including tissue-based protein/RNA markers as well as non-invasive monitoring tools based on liquid biopsy. Third, innovative clinical trials should be meticulously designed, such as "basket trials" or "platform trials" that use biomarker enrichment strategies to rapidly validate the efficacy of targeted strategies in small patient populations. Fourth, the combined application with existing therapies should continue to be expanded, particularly by combining ferroptosis/cuproptosis inducers with immune checkpoint inhibitors to leverage their ICD-inducing properties, thereby transforming "cold" tumors into "hot" tumors to overcome immunotherapy resistance [44, 80]. Finally, the development of novel, efficient, and tumor-targeting delivery systems (especially smart nanocarriers) is key to achieving clinical translation. Utilizing nanotechnology for the precise delivery and controlled release of metal ions, small-molecule drugs, or gene-editing tools, and integrating theranostic functions, holds the promise of enhancing therapeutic efficacy while minimizing systemic side effects [15, 27, 57].

## Data Availability

Not applicable.
